# Methods for handling longitudinal outcome processes truncated by dropout and death

**DOI:** 10.1093/biostatistics/kxx045

**Published:** 2017-09-26

**Authors:** Lan Wen, Graciela Muniz Terrera, Shaun R Seaman

**Affiliations:** 1 MRC Biostatistics Unit, University of Cambridge, IPH Forvie Site, Robinson Way, Cambridge, UK; 2 Centre for Dementia Prevention, University of Edinburgh, Kennedy Tower, UK

**Keywords:** Discrete-time independent censoring, Dropout, Generalized estimating equation, Imputation, Longitudinal data, Missing at random, Partly conditional inference

## Abstract

Cohort data are often incomplete because some subjects drop out of the study, and inverse probability weighting (IPW), multiple imputation (MI), and linear increments (LI) are methods that deal with such missing data. In cohort studies of ageing, missing data can arise from dropout or death. Methods that do not distinguish between these reasons for missingness typically provide inference about a hypothetical cohort where no one can die (immortal cohort). It has been suggested that inference about the cohort composed of those who are still alive at any time point (partly conditional inference) may be more meaningful. MI, LI, and IPW can all be adapted to provide partly conditional inference. In this article, we clarify and compare the assumptions required by these MI, LI, and IPW methods for partly conditional inference on continuous outcomes. We also propose augmented IPW estimators for making partly conditional inference. These are more efficient than IPW estimators and more robust to model misspecification. Our simulation studies show that the methods give approximately unbiased estimates of partly conditional estimands when their assumptions are met, but may be biased otherwise. We illustrate the application of the missing data methods using data from the ‘Origins of Variance in the Old–old’ Twin study.

## 1. Introduction

### 1.1. Motivation

Cohort studies involve measurements taken repeatedly over time, and studies with long follow-up often have missing data. A number of methods deal with missing data due to dropout in longitudinal studies (e.g. Schafer, 1997; Bang and Robins, 2005; Diggle *and others*, 2007, van Buuren and Groothuis-Oudshoorn, 2011), but not many describe how to handle missing data due to dropout and death (e.g. Kurland and Heagerty, 2005; Seaman *and others*, 2016).

We are motivated by the ‘Origins of Variance in the Old-Old’ (OCTO) Twin study. OCTO is a study of Swedish twins aged 80 years or older at recruitment, and it consists of five scheduled biennial visits. One continuous outcome measured in this study is peak expiratory function (henceforth, ‘lung function’) rate, which measures the maximal airflow at expiration, after maximal inspiration. In the older adults, lung function is associated with poor physical and cognitive health and mortality (Fragoso *and others*, 2007).

We assume a monotone missing data pattern, such as arises when subjects who drop out of the study do not return later. This is approximately true in the OCTO lung function data. In the OCTO study, 24% of the outcomes are missing due to death, and 27% are missing due to dropout, commonly because the subject had difficulty using the measuring instrument. Our goal is to estimate the expected lung function of survivors at each visit, and to understand how lung function is associated with smoking while subjects are alive. First, we describe estimands that may be of interest when outcomes are truncated by death.

### 1.2. Estimands

Suppose there are $N$ subjects in the study, and each subject has $J$ scheduled visits. Let $D$ be the last visit before the subject dies, regardless of whether the subject actually attended the visit. If $D={J}$, the subject is still alive at the end of the study. Let $Y_{j}$ be the continuous outcome of interest at visit $j$ ($j = 1, \ldots, J$).

Let $R_{j}$ denote the corresponding response indicator, i.e. $R_{j}=1$ if $Y_{j}$ is observed, and $R_{j}=0$ otherwise. Also let $\bar{Y}_{j} =(Y_{1}, \ldots, Y_{j})$, and $\bar{R}_{j} =(R_{1}, \ldots, R_{j})$. $Z = (Z_1, \ldots, Z_p)$ is a vector of $p$ fully observed baseline covariates. We assume $P(R_{1}=1)=1$.

Three estimands of interest are unconditional, partly conditional, and fully conditional on death (Kurland and Heagerty, 2005). These are parameters in models for, respectively, $E(Y_{j}|Z)$, $E(Y_{j}|Z, D\geq j)$, and $E(Y_{j}|Z, D)$.

When missingness is due to death, it is important to distinguish between inference for a mortal cohort and an immortal cohort. Immortal cohort inference makes no distinction between missingness due to death and missingness due to dropout. Unconditional estimands describe associations in immortal cohorts because the definition of $E(Y_{j}|Z)$ requires an implicit or explicit imputation of $Y_{j}$ for those subjects who die before $j$, thereby effectively creating a cohort that never dies (Dufouil *and others*, 2004). Since outcomes are undefined after death, $E(Y_{j}|Z, D< j)$, and therefore $E(Y_{j}|Z)$, is not meaningful. Hence, unconditional models (i.e. models for $E(Y_{j}|Z)$) are generally inappropriate when there is a non-negligible amount of missing data due to death (Kurland *and others*, 2009). Linear Mixed-effects Models (LMM) provide immortal cohort inference unless $D$ is included as a covariate or stratified on in the model.

Partly conditional models (i.e. models for $E(Y_{j}|Z, D \geq j)$) make inference about the expected outcome at a given time among survivors at that time point. Dufouil *and others* (2004) and Kurland and Heagerty (2005) favor partly conditional models. They argue that unconditional models would probably not be of interest unless the outcome and survival processes are independent. In this paper we focus on missing data methods that provide partly conditional inference.

Partly conditional models can be fitted using generalized estimating equations with independence working correlation matrix (IEE) when dropout among survivors at each time point is conditionally independent of the outcomes given $Z$, but may give biased estimates otherwise. To weaken this assumption, Kurland and Heagerty (2005) described the inverse probability weighted (IPW) method using IEE to weight up observed subjects to represent subjects who are still alive but have dropped out. This requires the estimation of the probability of dropout among subjects who are alive, given earlier outcomes and covariates.

Multiple imputation (MI; Schafer, 1997) is a commonly used method for handling missing data. However, the literature on how to use MI to handle dropout and death is limited (Harel *and others*, 2007), and this literature does not address the assumptions under which MI gives valid partly conditional inference.

Diggle *and others* (2007) introduced linear increments (LI) as a further method for handling missing data. This method allows the underlying outcome to be measured with an error that is independent of the covariates and the underlying outcome process. The LI methods developed by Aalen and Gunnes (2010) allows for non-monotone missing data, but does not allow for independent measurement error (Seaman *and others*, 2016). Other work on LI includes Gran *and others* (2017) who use LI to make causal inference, and Hoff *and others* (2014) who provide software in R to implement the LI method. Building on the work by Aalen and Gunnes (2010) and Seaman *and others* (2016) provide the underlying assumptions under which LI is a valid method for making partly conditional inference.

We have several aims in this article. In Section 3, we define and compare the assumptions of MI, LI, and IPW for making partly conditional inference. In particular, we show that, when we do not stratify on $D$ or include it as a covariate in the dropout or imputation model, the assumptions of MI and LI about the dropout and survival processes are different from those of IPW. In Section 4, we describe how to use MI, LI, and IPW when the imputation or dropout model stratifies on $D$ or includes it as a covariate. In Section 5, we illustrate graphically the assumptions described in Sections 3 and 4 using Directed Acyclic Graphs. In Section 6, we propose augmented IPW estimating equations for making partly conditional inference. These are attractive because they offer double protection against model misspecification. That is, they provide consistent estimations if the dropout or the imputation models are correctly specified. In Section 7, we provide simulation studies to compare bias and efficiency of the various missing data methods under various scenarios. Finally, in Section 8 we apply IPW, MI, and augmented IPW to data on lung function from the OCTO study. All proofs are in the supplementary material available at *Biostatistics* online (http://www.biostatistics.oxfordjournals.org).

## 2. Motivating example

**Fig. 1.**
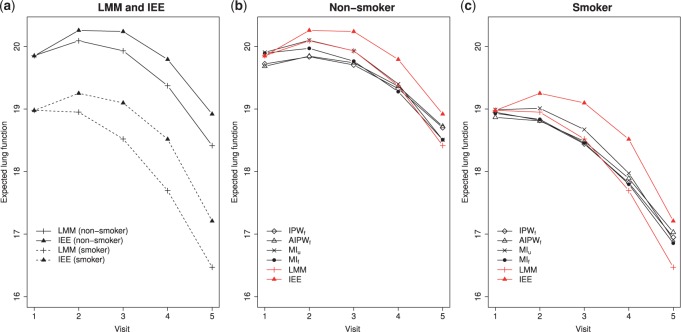
Expected lung function of men who were 83 years old at baseline with 7 years of education, stratified by smokers and non-smokers

As a motivating example, we model the association of lung function with time, age at baseline (age$_{{\rm base}}$), sex, education, smoking status, and the interactions between time and age$_{{{\rm base}}}$, sex, education, and smoking status in the OCTO study. Figure 1a shows the expected lung function of male smokers and non-smokers with average baseline age (83 years old) and average years of education (7 years). These means were estimated using both LMM and IEE. LMM do not distinguish between dropout and death, and IEE do not address dropout. In both smokers and non-smokers, the estimated means from LMM suggest a more rapid decline than do those from IEE. This is because unhealthier subjects (meaning those with poorer lung function) are more likely to die or drop out than are healthier subjects. Whereas IEE calculates the mean at each visit over only those subjects who have not yet dropped out or died (a healthier than average group), LMM calculates the mean over all subjects, implicitly imputing the (lower than average) missing lung functions. Moreover, because average lung function in smokers is lower than in non-smokers, there is more death in the smokers. Thus, the difference between the LMM and IEE estimates is greater for smokers than for non-smokers, with the result that LMM suggests smoking has a greater effect on rate of decline than does IEE. If dropout were independent of lung function, IEE would consistently estimate mean lung function conditional on still being alive. However, it is not. In the next two sections, we discuss various missing data methods that require weaker assumptions. A broad overview of these methods can be found in Table 1. Further discussion of this example can be found in Section 8.

**Table 1.** Assumptions and general guidelines for making partly conditional inference

Method	Assumption under which method is valid	Guidelines for use
IPW$_u$	u-MAR (1) u-MAR is equivalent to mortal-cohort dDTIC and missingness-independent death (2) u-MAR is the discrete death-time version of MAR in Kurland and Heagerty (2005)	Model probability of dropout at visit $j$ for those who survived up to visit $j$ Do not include $D$ in dropout models Appropriate when longitudinal and survival processes depend on one another, and survival process does *not* depend on dropout process u-MAR assumption can be tested if survival is known up to the end of study
IPW$_p$	p-MAR Note: p-MAR is a weaker assumption than u-MAR	Model probability of dropout at visit $k$ for those who survived up to and including visit $j$, $\forall k\leq j$
IPW$_f$	f-MAR (1) f-MAR is a weaker assumption than u-MAR (2) f-MAR is the discrete death-time version of MAR-S in Kurland and Heagerty (2005)	For visit each $j$, model the probability of dropout at visit $j$ for those who die between visits $j$ and $j+1$ (i.e. whose $D=j$) Include $D$ in dropout models
MI$_u$/LI$_u$	mortal-cohort dDTIC and independent death	Do not include $D$ in the imputation models Delete imputed post-death outcomes before analysis Appropriate when survival and dropout processes depend on one another, and survival process does *not* depend on missing outcome process
MI$_f$/LI$_f$	f-MAR	Include $D$ in imputation models Delete imputed post-death outcomes before analysis
AIPW$_u$	either i) u-MAR or ii) p-MAR, f-MAR and independent death	Do not include $D$ in dropout and imputation models Delete imputed post-death outcomes At least as efficient as IPW$_u$ if dropout and imputation models are correctly specified and survival process does not depend on longitudinal process (death still truncates longitudinal and dropout processes)
AIPW$_f$	f-MAR	Valid as long as either dropout or imputation models are correctly specified Include $D$ in dropout and imputation models Delete imputed post-death outcomes At least as efficient as IPW$_f$ if dropout and imputation models are correctly specified and data are f-MAR

## 3. Methods and assumptions

In this section, we describe how IPW, MI, and LI can be used to estimate the partly conditional estimand. We consider IPW, MI and LI where the dropout or imputation model do not stratify on $D$ or include it as a covariate, but these situations are the focus of Section 4.

### 3.1. Conditions under which MI produces consistent parameter estimates

Joint multivariate normal MI (Schafer, 1997) is one of the most popular methods for imputing missing data. It is widely available in many general statistical packages and is particularly suitable for handling missing continuous outcome variables. As we point out later, MI is closely related to LI when data are monotone missing.

Let $X$ be a vector that includes $Z$ and possibly also fully observed variables that are predictive of $Y$ (so-called ‘auxiliary variables’). Suppose that

(3.1) \begin{align*} & Y_{j} = \rho_{j} + \phi_{j}^T \bar{Y}_{j-1} + \psi_{j}^T X + \epsilon_{j}, \quad \forall j \leq D \label{eq:LI} \end{align*}

(3.2) \begin{align*} & \epsilon_{j}|\bar{Y}_{j-1}, X, D\geq j \sim \text{N}(0, \sigma_j^2) \label{eq:errorMI} \end{align*}

In MI, (3.1) and (3.2) are assumed to hold and the data are assumed to be missing at random (MAR). If no distinction is made between outcomes missing due to death and those missing due to other reasons, then all missing outcomes are imputed. Consequently, inference obtained using the imputed data will be for a ‘supplemented’ outcome process that consists of the actual pre-death outcomes and additional hypothetical post-death outcomes ($Y_{D+1}, \ldots, Y_J$). The pre-death outcomes are assumed to obey (3.1) and (3.2), and the hypothetical post-death outcomes are defined by $f(Y_j|\bar{Y}_{j-1},X, D<j) = f(Y_j|\bar{Y}_{j-1},X,D\geq j)$.

The joint distribution of $\bar{Y}_J$ in this supplemented process is $\bar{Y}_J|X\sim \text{N}(\mu, \Sigma)$, where $\mu$ and $\Sigma$ are functions of the $\rho_j$, $\phi_j$, $\psi_j$, and $\sigma_j^2$’s. In MI, given a non-informative prior distribution for $\theta=(\mu, \Sigma)$, missing outcomes in the supplemented process are drawn from their posterior predictive distribution. Conditional on $(\mu, \Sigma)$, if $\bar{Y}_{k}$ is observed but $(Y_{k+1},\ldots, Y_J)$ are missing, $(Y_{k+1},\ldots, Y_J)$ is sampled from the distribution $f(Y_{k+1},\ldots, Y_J|\bar{Y}_{k},X; \theta)$.

If a marginal model is fitted to all the imputed data including the post-death outcomes, an estimate of $E(Y_j \mid Z)$ for the supplemented process is obtained. To fit a partly conditional model, it is necessary to delete the imputed post-death outcomes and retain only the pre-death outcomes. The following two conditions are then sufficient for consistent estimation of parameters in the partly-conditional model for $j>k$: (1) $E(Y_{j}|\bar{Y}_k, R_k=1, R_{k+1}=0, X, D\geq j)$=$E(Y_{j}|\bar{Y}_k, X)$, and (2) $E(Y_{j}|\bar{Y}_k, X)$ is consistently estimated. Suppose that condition (2) is true, and $Y_k$ is the last observed outcome before visit $j$. Then the expected imputed value $\eta_j$ of $Y_j$ in a data set created by MI as $N\rightarrow \infty$, is equal to $Y_j$ if $R_j=1$ and $E(Y_{j}|\bar{Y}_k, X)$ if $R_j=0$.

If condition (1) is satisfied, then as shown by Seaman *and others* (2016), $E(\eta_j|Z, D\geq j)=E(Y_j|Z, D\geq j)$ and this ensures that the parameters of the partly conditional model are consistently estimated. To estimate these parameters, we exclude post-death outcomes from the imputed data sets, and use IEE to analyze each.

Condition (1) is satisfied if the conditional distribution of the missing outcomes in the supplemented process given the earlier observed outcomes and covariates is the same whether or not the missing outcomes are after death, i.e. if

(3.3) \begin{align*} f(Y_{k+1}, \ldots, Y_j|\bar{Y}_{k},R_{k}=1,R_{k+1}=0,X, D\geq j)=f(Y_{k+1},\ldots, Y_j|\bar{Y}_{k},X), \forall j\geq k+1 \label{eq:mdid} \end{align*}

Seaman *and others* (2016) proved that if (3.1) and (3.2) hold, and the two assumptions specified below (mortal-cohort dDTIC and independent death) are satisfied, then (3.3) (and hence condition (1)) holds. It can then be shown that these assumptions imply that the data on the supplemented process are MAR. This then implies that condition (2) holds.

*Mortal-cohort discrete-time independent censoring in distribution* (*dDTIC*) is defined by Seaman *and others* (2016) as

(3.4) \begin{equation*}f(Y_{j}|\bar{Y}_{j-1}, \bar{R}_{j}, X, D\geq j) = f(Y_{j}|\bar{Y}_{j-1}, X, D\geq j), \forall j \label{eq:mortal} \end{equation*}

which is equivalent to $P(\bar{R}_{j}|\bar{Y}_{j}, X, D\geq j) = P(\bar{R}_{j}|\bar{Y}_{j-1}, X, D\geq j)$, i.e. conditional on survival up to visit $j$ and outcomes prior to visit $j$, the missingness history up and including visit $j$ does not depend on the outcome at visit $j$. Note that mortal cohort dDTIC looks similar to the classical MAR assumption conditional on subjects alive. However, in Appendix B of supplementary material available at *Biostatistics* online, we show that it is not the same.

The *independent death* assumption is defined by Seaman *and others* (2016) as, $\forall j>k$:

(3.5) \begin{align*} P(D \geq j|D \geq j-1,R_{k}=1, R_{k+1}=0, \bar{Y}_{j-1},X)=P(D \geq j|D \geq j-1, R_{k}=1, R_{k+1}&=0, \bar{Y}_{k},X) \label{eq:indptdeath} \end{align*}

This assumption says the probability of dying between visits $j-1$ and $j$ for people who attended visit $k$, but not visit $k+1$, could depend on the past observed outcomes, but not on any subsequent unobserved outcomes.

In summary, MI is a valid method for making partly conditional inference if (3.1) and (3.2) hold, mortal-cohort dDTIC and independent death are satisfied, and post-death outcomes are deleted before analyzing each imputed data set using IEE.

MI assumes that $\epsilon_j$ is normally distributed as described by (3.2). However, as shown in Theorem 4 of Seaman *and others* (2016), when data are monotone missing, this assumption is stronger than required for consistent estimation of the parameters in the partly conditional model. Instead, the following weaker condition is sufficient:

(3.6) \begin{equation*} E(\epsilon_{j}|\bar{Y}_{j-1}, X, D\geq j)=0, \text{and Var}(\epsilon_j|\bar{Y}_{j-1},X,D\geq j )=\text{Var}(\epsilon_j|D\geq j) < \infty \label{eq:error} \end{equation*}

Equation (3.2) is needed, though, for Rubin’s rules (Schafer, 1997) to give asymptotically unbiased estimation of the variance of the parameter estimators. Bootstrapping can be used to estimate this variance when (3.6) but not (3.2) holds.

### 3.2. A comparison with LI

For monotone missing data, LI imputation (Seaman *and others*, 2016) is asymptotically equivalent to MI with an infinite number of imputations. As with MI, the LI imputation method provides consistent parameter estimates of a model for $E(Y_j|Z, D\geq j)$ provided that (3.1) and (3.6) hold, mortal-cohort dDTIC and independent death hold, and post-death imputed outcomes are deleted before analyzing the imputed data using IEE (Seaman *and others*, 2016). Details about LI imputation are provided in Appendix A of supplementary material available at *Biostatistics* online.

### 3.3. Conditions under which IPW produces consistent parameter estimates

Let $\mu_j =\mu_j(Z)= E(Y_j|D\geq j,Z)$. A subject’s contribution to the IEE is

\begin{align*}
\sum_{j=1}^J Z_j \text{I}(D \geq j)R_{j}(Y_{j}-\mu_{j})
\end{align*}

and the $j$th element of the estimating equations is $Z_j\text{I}(D\geq j)R_{j}(Y_{j}-\mu_{j})$. In IPW, we multiply the $j$th element of the estimating equation by the inverse of $P(R_{j}=1|D\geq j, \bar{Y}_{j},X)$. Since the probability $P(R_{j}=1|D\geq j, \bar{Y}_{j},X)$ is unknown, it needs to be modeled. Let $\pi_j(\tau)$ be a model for $P(R_j=1|\bar{Y}_{j}, D\geq j,X)$, with associated parameters $\tau$. The $j$th element of the estimating equations becomes

\begin{align*}
\frac{Z_j\text{I}(D \geq j)R_{j}}{\pi_j(\tau)}(Y_{j}-\mu_{j})
\end{align*}

Dufouil *and others* (2004) provide non-algebraic descriptions of the assumptions of the IPW method. First, they say that they assume the “probability that [an outcome] is missing may depend on other observed parts of the response profile, but does not depend on unobserved [outcomes].” Second, they say that they assume the “mortality rates following dropout from the study are the same as the corresponding rates for subjects remaining in the study.” The first assumption is ambiguous because it appears to be describing MAR, which does not distinguish between subjects who are alive and dead, thereby making the assumption hard to interpret. The second assumption is ambiguous because it does not specify whether the mortality rates are conditional or unconditional on any of the past outcomes.

Kurland and Heagerty (2005) provide algebraic expressions for the assumptions of IPW and introduce three ways to model $P(R_{j}=1|D\geq j, \bar{Y}_{j},X)$ when the missingness depends on some of the past observed outcomes.

Neither Dufouil *and others* (2004) nor Kurland and Heagerty (2005) compare the assumptions of the various IPW methods or compare these methods with alternative methods. In this section and the next, we expand on their work by distinguishing the IPW assumptions and their corresponding estimators, and compare these assumptions to those of MI and LI imputation.

We distinguish between two MAR-type assumptions: *unconditional MAR* (*u-MAR*) and *partly-conditional MAR* (*p-MAR*). *u-MAR* is the assumption that

(3.7) \begin{equation*}P(R_{k} = 1| R_{k-1}=1 , \bar{Y}_{j}, D=j,X) = P(R_{k} = 1| R_{k-1}=1 , \bar{Y}_{k-1}, D \geq k,X) \forall k\leq j \label{eq:markh2}\end{equation*}

which is equivalent to $P(R_{k}=1| D=j, \bar{Y}_{j},X) = P(R_{k}=1|D\geq k, \bar{Y}_{k-1},X)$, $\forall k\leq j$. Under u-MAR, the probability of observing the outcome at visit $j$ given survival up to that time is

\[P(R_{j} = 1|\bar{Y}_{j}, D\geq j,X) = \prod_{k=1}^j P(R_{k} = 1|R_{k-1}=1 , \bar{Y}_{k-1}, D\geq k,X)\]

We can compare this assumption with the assumptions of MI and LI imputation. To do this, we define *missingness-independent death* as

(3.8) \begin{equation*}P(D\geq j|D\geq j-1, \bar{Y}_{j-1}, \bar{R}_{j-1},X) = P(D\geq j|D\geq j-1, \bar{Y}_{j-1},X), \forall j\geq 3\label{eq:missindptdeath}\end{equation*}

In contrast to independent death, which says that the probability of dying between visits $j-1$ and $j$ does not depend on the past missing outcomes given the past observed outcomes and *the time of dropout*, missingness-independent death says that the probability of dying between visits $j-1$ and $j$ does not depend on the past missingness history given *all* of the *past outcomes*.

Theorem 1 u-MAR holds if and only if mortal-cohort dDTIC and missingness-independent death both hold.

*p-MAR* is the assumption that

(3.9) \begin{equation*} P(R_{k} = 1|R_{k-1}=1 , \bar{Y}_{j}, D\geq j,X) = P(R_{k} = 1|R_{k-1}=1 , \bar{Y}_{k-1}, D\geq j,X) \forall k\leq j \label{eq:MAR2} \end{equation*}

or equivalently, $P(R_{k} = 1|\bar{Y}_{j}, D\geq j,X) = P(R_{k} = 1| \bar{Y}_{k-1}, D\geq j,X)$, $\forall k\leq j$. Under p-MAR, the probability of observing the outcome at visit $j$ given survival up to that time is

\[P(R_{j} = 1|\bar{Y}_{j}, D\geq j,X) = \prod_{k=1}^j P(R_{k} = 1|R_{k-1}=1, \bar{Y}_{k-1}, D\geq j,X).\]

We might prefer to assume p-MAR rather than u-MAR because it is a weaker assumption than u-MAR. However, the IPW method based on the p-MAR assumption requires the survival statuses to be known up to the end of a study in order to fit models for $P(R_{k} = 1|R_{k-1}=1 , \bar{Y}_{j}, D\geq j,X)$, whereas the IPW method based on the u-MAR assumption requires the survival statuses only to be known up to and including the time of dropout.

## 4. Methods fully conditional on death

### 4.1. IPW

If times of all deaths occurring prior to the end of the study are known, we could instead assume *fully conditional MAR* (*f-MAR*):

(4.1) \begin{equation*} P(R_{k}=1|R_{k-1}=1,\bar{Y}_{j},D=j,X) = P(R_{k}=1|R_{k-1}=1,\bar{Y}_{k-1},D=j,X) \forall k\leq j \label{eq:dropoutMARD} \end{equation*}

or equivalently, $P(R_{k}=1|\bar{Y}_{j},D=j,X) = P(R_{k}=1|\bar{Y}_{k-1},D=j,X)$, $\forall k\leq j$. Under f-MAR, the probability of observing the outcome at visit $j$ can be written as

(4.2) \begin{equation*} P(R_{j}=1|\bar{Y}_{j},D=l,X) = \prod_{k=1}^j P(R_{k} = 1|R_{k-1}=1 , \bar{Y}_{k-1}, D=l,X) \forall l\geq j \label{eq:MARD}\end{equation*}

u-MAR is a stronger assumption than p-MAR and f-MAR, so the IPW method that relies on the u-MAR assumption (henceforth called IPW$_u$) may be more efficient when this assumption is true than the IPW methods that rely on the p-MAR and f-MAR assumptions (henceforth called IPW$_p$ and IPW$_f$, respectively). Moreover, for IPW$_u$, survival statuses only need to be known up to and including the time of dropout, but for IPW$_p$ and IPW$_f$, survival statuses need to be known until the end of a study. Note that neither p-MAR nor f-MAR implies the other.

### 4.2. MI and LI

For MI and LI imputation, instead of assuming (3.1) and (3.2), suppose that for those subjects with $D=l$,

(4.3) \begin{equation*} Y_{j} = \rho_{j}^{(l)} + (\phi_{j}^{(l)})^T \bar{Y}_{j-1} + (\psi_{j}^{(l)})^T X + \epsilon_{j}^{(l)} \quad \forall j \leq l \label{eq:LID} \end{equation*}

with

(4.4) \begin{equation*} \epsilon_j^{(l)}|\bar{Y}_{j-1},X,D=l \sim \text{N}[0, (\sigma_j^{(l)})^2] \label{eq:errorD} \end{equation*}

The vector of outcomes $({Y}_1,\ldots, Y_l)$ then has the joint distribution $\bar{Y}_l|X,D=l \sim \text{N}(\mu_j^{(l)},\Sigma_j^{(l)})$, where $\mu_j^{(l)}$ and $\Sigma_j^{(l)}$ are functions of $\rho_{j}^{(l)}$, $\phi_{j}^{(l)}$, $\psi_{j}^{(l)}$, and $\sigma_{j}^{(l)}$’s. Under (4.3)–(4.4) and f-MAR, if visit $k$ is the last visit attended before visit $j$, then as $N\rightarrow \infty$, the expected imputed value of $Y_j$ in a data set created by MI will be $E(Y_j|\bar{Y}_k,X,D=l)$ when $R_j$=0. As shown in Appendix H of supplementary material available at *Biostatistics* online, it is then valid to use the imputed data to estimate the partly conditional mean.

Henceforth, we will call MI and LI imputation based on (3.1) and (3.2) MI$_u$ and LI$_u$ imputation, respectively. We will call MI and LI imputation based on (4.3) and (4.4) MI$_f$ and LI$_f$ imputation, respectively.

Instead of stratifying on $D$ (as in (4.3) and (4.4)), we can include it as a covariate in the imputation model. Doing this may be a more feasible option when the sample size in at least some of the strata defined by $D$ is small.

## 5. Directed Acyclic Graphs depicting scenarios for the missing data methods

### 5.1. Graph 1: most complex scenario for u-MAR to hold

**Fig. 2.**
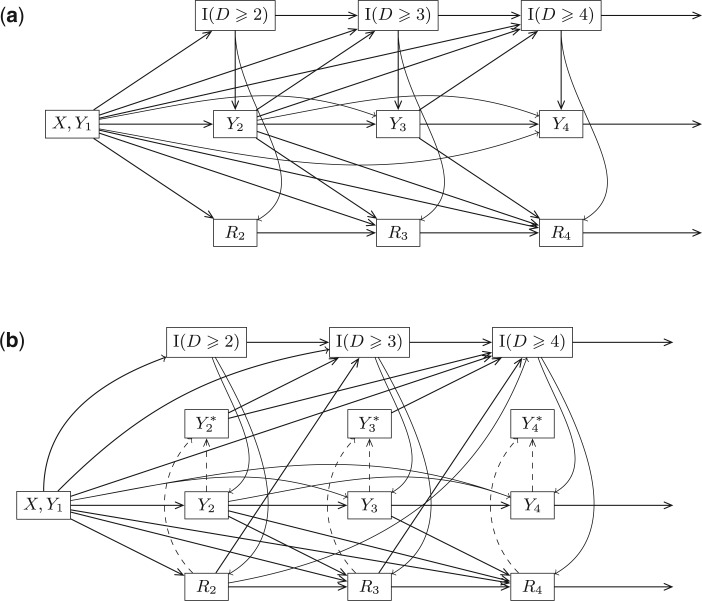
Directed Acyclic Graphs for scenarios 1 and 2. (a) Directed Acyclic Graph 1 for scenario 1 (Most complex scenario for u-MAR to hold). (b) Directed Acyclic Graph 2 for scenario 2 (Most complex scenario for mortal cohort dDTIC and independent death to hold). Dashed lines represent deterministic associations (e.g. $Y_2^*$ is determined by $R_2$ and $Y_2$).

The Directed Acyclic Graphs in this section describe data generating mechanisms where the survival statuses, the longitudinal outcomes, and the dropout statuses are generated in a temporal order. For these graphs, we define $Y_j=\varnothing$ when $D<j$. Graph 1 (Figure 2a) shows the most complex scenario where u-MAR holds. It is the most complex scenario because adding a directed edge of the form $R_j \rightarrow Y_{j+1}$ to Graph 1 would allow the mechanism to violate mortal-cohort dDTIC, and adding a directed edge $R_j \rightarrow \text{I}(D\geq j+1)$ would allow it to violate missingness-independent death. When u-MAR holds, IPW$_{u}$, IPW$_{p}$, and IPW$_{f}$ give consistent estimates, provided that the dropout models are correctly specified (Section 3.3).

Graph 1 allows the data generating mechanism to violate independent death, and so MI$_u$ and LI$_u$ imputation may not provide consistent estimates under mechanisms described by Graph 1. However, u-MAR implies f-MAR. Consequently, MI$_f$ or LI$_f$ imputation would give consistent estimates, provided that the imputation models were correctly specified (Section 4.2).

### 5.2. Graph 2: most complex scenario for mortal-cohort dDTIC and independent death to hold

Let $Y_j^* = Y_j$ if $R_j=1$ and $Y_j^*=\varnothing$ if $R_j=0$, and let $\bar{Y}_j^* = (Y_1^*, \ldots,Y_j^*)$. So, if $R_k=1$, $R_{k+1}=0$ and $j>k$, then $\bar{Y}_{j-1}^*=(Y_1,\ldots,Y_k, \varnothing, \ldots, \varnothing)$. Independent death can now be written as

\[P(D \geq j|D \geq j-1, R_{k}=1, R_{k+1}=0, \bar{Y}_{j-1},X) = P(D \geq j|D \geq j-1, R_{k}=1, R_{k+1}=0, \bar{Y}_{j-1}^*,X)\]

Introducing $Y_j^*$ allows us to draw a Directed Acyclic Graph that satisfies independent death. Graph 2 (Figure 2b) is the most complex scenario under which LI$_u$ imputation and MI$_u$ provide consistent estimates. This is because adding a directed edge $R_j \rightarrow Y_{j+1}$ would allow violation of mortal-cohort dDTIC, and adding a directed edge $Y_j \rightarrow \text{I}(D\geq j+1)$ would allow violation of independent death.

Graph 2 fails to satisfy missingness-independent death, and so, by Theorem 1, it fails to satisfy u-MAR. Hence, estimates from IPW$_u$ may be biased. Graph 2 also fails to satisfy p-MAR and f-MAR, because, for example, the directed edges $Y_2^* \rightarrow \text{I}(D\geq 3)$ induce an association between $R_2$ on $Y_2$ conditional on $\{D\geq 3\}$, ${Y}_{1}$ and $X$ (as shown in Appendix C of supplementary material available at *Biostatistics* online). Therefore, estimates from IPW$_p$, IPW$_f$, MI$_f,$ and LI$_f$ imputation may be biased.

## 6. Augmented IPW

Robins, Rotnitzky, and colleagues introduced the augmented IPW (AIPW) method in a series of seminal papers including Robins *and others* (1995) and Scharfstein *and others* (1999). AIPW methods require specification of a model for the probability of dropout *and* an imputation model for the expectation of each missing outcome. The AIPW method is attractive because it provides consistent parameter estimates as long as one of these two models is correctly specified.

Bang and Robins (2005) and Seaman and Copas (2009) described AIPW to handle longitudinal data that are monotone missing due to dropout. We now adapt this method to handle missingness due to dropout and death. Like the IPW method for partly-conditional inference, this method requires an independence working correlation matrix.

Let $\mu_j =E(Y_j|Z,D\geq j)= {\beta}^TZ$, where $ {\beta}$ is a vector of parameters of interest, and assume that $D$ is included in $X$. For $j\in\{1, \ldots, J\}$, let $H_j(\bar{Y}_j, X; {\beta} , {\gamma})$ be a model for $E[U(\beta)|\bar{Y}_{j},X, R_j=1]$, where $U(\beta)=U(\bar{Y}_D,D, Z;\beta)=\sum_{j=1}^D Z_j(Y_j-\mu_j)$. $H_j(\bar{Y}_j, X; {\beta} , {\gamma})$ can be any function of $\bar{Y}_j$, $X$ and parameters $ {\gamma}$ that obeys $H_D(\bar{Y}_D, X; {\beta} , {\gamma})=U(\beta)$. Let $\tilde{\pi}_j (\bar{Y}_{j-1}, X; {\alpha})$ be a model for $P(R_j=1|\bar{Y}_{j-1}, X)$ with parameters $ {\alpha}$. Let $ {\hat{\gamma}}$ and $ {\hat{\alpha}}$ denote consistent estimators of $ {\gamma}$ and $ {\alpha}$. A subject’s contribution to the AIPW estimating equations for $ {\beta}$ is,

(6.1) \begin{equation*} \Psi( { {\beta}}, {\hat{\alpha}}, {\hat{\gamma}}) = \left[\frac{R_{D}}{\tilde{\pi}_{D}(\bar{Y}_{D-1}, X; {\hat{\alpha}})} U( {\beta}) + \sum_{j=1}^{D-1} \left\{\frac{R_{j}}{\tilde{\pi}_{j}(\bar{Y}_{j-1}, X; {\hat{\alpha}})} - \frac{R_{j+1}}{\tilde{\pi}_{j+1}(\bar{Y}_{j}, X; {\hat{\alpha}})} \right\} H_j(\bar{Y}_j, X; {\beta} , {\hat{\gamma}})\right] \label{eq:aipw} \end{equation*}

The resulting estimators of $ {\beta}$ will be consistent if either $\tilde{\pi}_j(\bar{Y}_{j-1}, X;\alpha)$ is correctly specified for all $j$ or $H_j(\bar{Y}_j, X; {\beta} , {\gamma})$ is correctly specified for all $j$. If both models are correctly specified, this AIPW estimator is at least as efficient and usually more efficient than the IPW$_f$ estimator.

Theorem 2 If the data are f-MAR, the AIPW gives consistent estimations if either $\tilde{\pi}_j(\bar{Y}_{j-1}, X;\alpha)$ or $H_j(\bar{Y}_j, X; {\beta} , {\gamma})$ is correctly specified. Moreover, consistent estimates of $ {\beta}$ are still obtained when the models for $\tilde{\pi}_j(\bar{Y}_{j-1}, X;\alpha)$ and $H_j(\bar{Y}_j, X; {\beta} , {\gamma})$ omit the covariate $D$ (or do not stratify on $D$) provided that either (1) u-MAR holds and the dropout model is correctly specified, or (2) f-MAR, p-MAR and independent death hold and $H_j(\bar{Y}_j, X; {\beta} , {\gamma})$ is correctly specified.

When models $\tilde{\pi}_j(\bar{Y}_{j-1}, X;\alpha)$ and $H_j(\bar{Y}_j, X; {\beta} , {\gamma})$ stratify on $D$ or include $D$ as a covariate, the AIPW method will be called AIPW$_f$. When $\tilde{\pi}_j(\bar{Y}_{j-1}, X;\alpha)$ and $H_j(\bar{Y}_j, X; {\beta} , {\gamma})$ exclude $D$, it will be called AIPW$_u$. Note that if the data generating mechanism satisfies u-MAR and independent death, then AIPW$_u$ gives consistent estimations if either $\tilde{\pi}_j(\bar{Y}_{j-1}, X;\alpha)$ or $H_j(\bar{Y}_j, X; {\beta} , {\gamma})$ is correctly specified. We can use logistic regression to model dropout and Paik’s mean imputation (Paik, 1997) to estimate the conditional expectation of each missing outcome.

Under the data generating mechanism described by Directed Acyclic Graph 1, we expect AIPW$_{u}$ to yield consistent $ {\beta}$ estimates provided that the dropout models are correctly specified, and AIPW$_{f}$ to yield consistent $ {\beta}$ estimates provided that the dropout or the imputation models are correctly specified. However, under the data generating mechanism described by Directed Acyclic Graph 2, we expect AIPW$_{u}$ and AIPW$_{f}$ to yield inconsistent $ {\beta}$ estimates in general.

## 7. Simulation study

### 7.1. Methods

In the following simulation study, we compare the different assumptions underlying the missing data methods. The baseline covariate is generated and the parameters for the longitudinal, survival, and dropout models are chosen to mimic the data from the OCTO study.

We compare the bias, standardized bias and efficiency of the missing data methods. Standardized bias is $100\times$[(Average estimate$-$True parameter)/empirical standard deviation of the parameter estimates]. It has been suggested that an absolute standardized bias of approximately 40% will be “practically significant” and will have a “noticeable adverse impact on efficiency, coverage, and error rates.” (Collins *and others*, 2001).

Simulations 1 and 2 correspond to simplified versions (shown in Figure D.1 in the Appendix D of supplementary material available at *Biostatistics* online) of Directed Acyclic Graphs 1 and 2. In the simplified version of Graph 1, $Y_j$, $R_j$, and $I(D\geq j)$ depend on $Y_{j-1}$, but not on $\bar{Y}_{j-2}$. In the simplified version of Graph 2, $Y_j$ and $R_j$ depend on $Y_{j-1}$, but not on $\bar{Y}_{j-2}$; $I(D\geq j)$ depends on the last *observed* outcome, but not on the ones before. In both of the simulations, data are generated in a sequential manner. For example, under simulation 1, data are generated by:

1. $Y_1$ from $f(Y_1)$ and $X$ from $f(X)$
2. $I(D\geq 2)$ from $P(D\geq 2| D\geq 1,Y_1,X)$
3. $R_2$ from $P(R_{2}=1|R_{1}=1, D\geq 2, Y_1,X)$, and $P(R_{2}=1|R_{1}=1, D<2, Y_1,X)=0$
4. $Y_2$ from $f(Y_2|Y_1, D\geq 2,X)$ ($Y_2$ is not generated for those with $D<2$)

and so on. More details are given in Appendix D of supplementary material available at *Biostatistics* online. Approximately 24% of outcomes are missing due to death, and of those who are alive at each visit, approximately 27% are missing due to dropout. The analysis model we use for the simulations is

(7.1) \begin{align*} E(Y_{j}|{{\rm sex}}, D\geq j) & = \beta_{0} + \beta_{1}\text{I}(j=1)+\beta_{2}\text{I}(j=2)+\beta_{3}\text{I}(j=3)+\beta_{4}\text{I}(j=4) + \beta_{{{\rm sex}}} {{\rm sex}} + \nonumber \\ & \beta_{{{\rm sex}}2}\text{I}(j=2) \cdot {{\rm sex}}+\beta_{{{\rm sex}}3}\text{I}(j=3) \cdot {{\rm sex}}+\beta_{{{\rm sex}}4}\text{I}(j=4) \cdot {{\rm sex}} \label{eq:analysissim} \end{align*}

For each simulation, we generate 1000 data sets, assume $J=5$, $N=500$ or 1000, and use 30 imputations in each MI procedure. The correct dropout and imputation models are used, with the exception of the AIPW$_f$ method in simulation 1. In simulation 1, we show that AIPW$_f$ are doubly robust against model misspecification by using a misspecified dropout or a misspecified imputation model (omitting sex in one of the dropout and imputation models).

### 7.2. Results

In both simulation studies, IEE and complete case (subjects observed at all five visits) analysis overestimate the average outcomes in males and females at all visits. Table 2a and Table J.4 in Appendix J of supplementary material available at *Biostatistics* online, show, for 500 and 1000 subjects, the bias, standardized bias, and the empirical standard error for simulation 1. The biases from IPW$_{u}$, IPW$_{p}$, IPW$_{f}$, AIPW$_{u}$, AIPW$_{f}$, LI$_f$ imputation, and MI$_f$ are negligible, but the biases from LI$_u$ imputation and MI$_u$ are bigger and/or practically significant. This is because, as explained in Section 5.1, Graph 1 fails to satisfy independent death.

**Table 2.** Results from simulation 1 and 2 (N=500). In the imputation and dropout models of method (1), $D$ is modeled as a covariate, and in the imputation and dropout models of method (2), $D$ is stratified on.

(a) Bias ($\times 100$), standardized bias (s-bias) and standard error ($\times 100$) from simulation 1																								
Parameter	$\beta_1=0.698$			$\beta_2=0.962$			$\beta_3=0.567$			$\beta_4=-0.162$			$\beta_{{\rm sex}1}=-0.535$			$\beta_{{\rm sex}2}=-1.087$			$\beta_{{\rm sex}3}=-1.673$			$\beta_{{\rm sex}4}=-1.248$		
	bias	s-bias	SE	bias	s-bias	SE	bias	s-bias	SE	bias	s-bias	SE	bias	s-bias	SE	bias	s-bias	SE	bias	s-bias	SE	bias	s-bias	SE
CC$^\dagger$	30.7	101.0	11.6	5.4	13.8	16.7	$-$14.0	$-$32.4	21.2	$-$64.1	$-$130.8	27.2	$-$8.1	$-$20.8	16.4	$-$8.0	$-$15.9	22.9	4.0	7.2	28.0	12.0	19.3	35.1
IEE$^\ddagger$	16.1	97.9	16.4	31.7	134.7	23.5	34.6	109.7	31.5	44.6	105.3	42.3	1.5	6.5	23.0	$-$5.2	$-$15.7	33.5	$-$6.7	$-$16.3	41.2	$-$4.5	$-$8.3	54.7
IPW$_u$$^\ddagger$	$-$0.3	$-$1.6	16.0	$-$0.7	$-$3.0	23.2	$-$0.8	$-$2.4	32.5	0.1	0.3	46.3	0.1	0.5	23.2	0.4	1.3	34.8	1.0	2.4	44.0	1.0	1.7	60.8
IPW$_p$$^\ddagger$	$-$0.3	$-$1.6	16.0	$-$0.8	$-$3.3	23.2	$-$0.9	$-$2.7	32.4	0.5	1.0	46.4	0.1	0.5	23.2	0.5	1.3	34.9	1.1	2.5	44.1	0.8	1.3	61.2
IPW$_f$ (1)$^\ddagger$	$-$0.3	$-$1.9	15.9	$-$0.8	$-$3.2	23.3	$-$0.8	$-$2.4	32.6	0.1	0.3	46.3	0.1	0.5	23.2	0.5	1.3	34.8	1.0	2.3	44.0	1.0	1.7	60.9
IPW$_f$ (2)$^\ddagger$	$-$0.4	$-$2.3	15.7	$-$1.0	$-$4.0	23.8	$-$0.6	$-$2.0	32.9	0.5	1.0	46.4	0.1	0.3	22.7	0.6	1.7	35.1	0.8	1.8	44.5	0.8	1.3	61.2
AIPW$_u$$^\ddagger$	$-$0.2	$-$1.5	15.2	$-$1.1	$-$5.1	22.2	$-$1.3	$-$4.2	30.1	$-$1.3	$-$2.9	43.1	0.1	0.3	21.2	0.9	3.0	32.0	1.5	3.8	40.0	2.2	3.9	55.5
AIPW$_f$(1)$^\ddagger$	$-$0.3	$-$1.7	15.1	$-$1.3	$-$5.8	22.2	$-$1.4	$-$4.8	29.8	$-$0.9	$-$2.1	43.2	0.0	0.2	21.0	1.0	3.0	31.8	1.6	4.1	39.8	1.9	3.4	55.6
AIPW$_f$(2)$^\ddagger$	$-$0.3	$-$2.0	15.1	$-$1.3	$-$5.8	22.6	$-$1.3	$-$4.2	30.2	$-$1.0	$-$2.3	43.2	0.0	0.0	21.0	0.9	2.7	32.2	1.4	3.6	40.4	2.0	3.6	55.7
LI$_u$$^\ddagger$	$-$0.3	$-$1.6	15.3	$-$4.4	$-$20.2	21.8	$-$10.5	$-$36.5	28.7	$-$17.8	$-$43.9	40.5	0.1	0.5	21.2	1.1	3.5	31.0	4.8	12.6	37.9	7.0	13.6	51.4
LI$_f$(1)$^\ddagger$	$-$0.4	$-$2.5	15.1	$-$2.3	$-$10.7	21.8	$-$1.0	$-$3.6	28.9	$-$1.6	$-$3.9	40.8	0.2	0.8	21.0	1.5	4.9	30.8	1.6	4.2	37.9	2.4	4.7	51.4
LI$_f$(2)$^\ddagger$	$-$0.4	$-$2.9	15.2	$-$1.7	$-$7.8	22.0	$-$1.5	$-$5.1	29.7	$-$1.4	$-$3.4	41.4	0.2	0.8	21.1	1.4	4.6	31.2	1.8	4.5	39.0	2.4	4.6	52.4
MI$_u$$^\ddagger$	$-$0.4	$-$2.9	15.2	$-$5.3	$-$24.3	21.9	$-$12.0	$-$41.7	28.8	$-$19.7	$-$48.7	40.4	0.1	0.4	21.0	1.2	3.8	31.2	5.0	13.2	38.1	7.3	14.2	51.4
MI$_f$(1)$^\ddagger$	$-$0.6	$-$4.0	15.2	$-$1.7	$-$7.8	21.8	1.3	4.5	29.0	1.7	4.2	41.0	0.4	1.7	21.1	1.7	5.5	30.8	1.3	3.5	37.9	1.6	3.2	51.5
MI$_f$(2)$^\ddagger$	$-$0.3	$-$2.2	15.2	$-$2.4	$-$10.8	22.1	$-$3.1	$-$10.4	29.8	$-$3.0	$-$7.3	41.4	0.2	0.9	21.1	1.6	5.1	31.4	1.9	4.7	39.1	2.6	4.9	52.6
$^\dagger$bias, standardized bias, and standard error for CC (complete case): ${\beta}_0 = (108.1,439.8,24.6)$ and ${\beta}_{{\rm sex}} =(-16.3,-50.5,32.3).$																								
$^\ddagger$bias, standardized bias, and standard error for all other methods: ${\beta}_0 = (0.0,0.3,14.5)$ and ${\beta}_{{\rm sex}} =(0.3,1.5,21.0)$.																								
(b) Bias ($\times 100$), standardized bias (s-bias) and standard error ($\times 100$) from simulation 2																								
Parameter	$\beta_1=0.698$			$\beta_2=0.872$			$\beta_3=0.354$			$\beta_4=-0.414$			$\beta_{{\rm sex}1}=-0.535$			$\beta_{{\rm sex}2}=-1.095$			$\beta_{{\rm sex}3}=-1.616$			$\beta_{{\rm sex}4}=-1.232$		
	bias	s-bias	SE	bias	s-bias	SE	bias	s-bias	SE	bias	s-bias	SE	bias	s-bias	SE	bias	s-bias	SE	bias	s-bias	SE	bias	s-bias	SE
CC$^\S$	25.7	92.0	11.6	7.2	20.5	16.7	0.9	2.2	21.2	$-$39.9	$-$88.9	27.2	$-$5.9	$-$16.2	16.4	$-$5.5	$-$12.1	22.9	$-$1.9	$-$3.7	28.0	6.8	11.9	35.1
IEE^¶^	15.7	97.5	16.1	37.0	159.7	23.2	50.7	169.4	29.9	59.7	153.2	38.9	2.4	10.7	22.4	$-$4.1	$-$12.8	32.0	$-$13.6	$-$34.0	40.0	$-$9.7	$-$19.1	50.7
IPW$_u$^¶^	$-$0.9	$-$5.5	15.6	3.1	13.0	23.6	13.3	42.2	31.5	11.5	28.1	41.0	1.1	5.0	22.7	2.0	6.0	32.9	$-$5.4	$-$12.7	42.4	$-$4.1	$-$7.4	55.9
IPW$_p$^¶^	$-$0.9	$-$5.5	15.6	2.3	9.9	23.7	11.6	36.2	32.1	18.5	44.7	41.4	1.1	5.0	22.7	$-$0.2	$-$0.7	33.0	$-$10.4	$-$23.9	43.7	$-$14.3	$-$25.1	57.0
IPW$_f$ (1)^¶^	$-$1.3	$-$8.5	15.8	$-$17.4	$-$62.9	27.7	3.2	8.3	38.4	24.8	63.4	39.0	1.0	4.6	22.7	9.4	25.0	37.5	$-$12.1	$-$22.9	52.6	$-$10.0	$-$18.8	53.4
IPW$_f$ (2)^¶^	$-$3.6	$-$21.7	16.4	3.1	12.9	23.9	1.6	4.4	36.1	18.5	44.7	41.4	3.6	15.7	23.3	$-$7.3	$-$21.3	34.4	$-$8.0	$-$16.7	47.9	$-$14.3	$-$25.1	57.0
AIPW$_u$^¶^	$-$0.8	$-$5.3	14.8	1.9	8.7	22.3	9.2	31.2	29.6	18.5	46.1	40.2	1.0	4.9	20.5	0.4	1.5	30.3	$-$5.0	$-$12.8	39.0	$-$12.2	$-$23.6	51.7
AIPW$_f$ (1)^¶^	$-$1.9	$-$13.0	14.9	$-$2.3	$-$9.7	23.8	$-$3.7	$-$11.7	31.3	14.2	38.0	37.5	3.1	15.1	20.4	3.4	10.5	32.3	1.3	3.2	41.8	$-$8.2	$-$16.9	48.5
AIPW$_f$ (2)^¶^	$-$4.7	$-$30.2	15.5	$-$1.5	$-$6.6	23.0	$-$3.2	$-$9.8	32.8	14.8	37.8	39.2	5.6	26.8	21.0	2.2	6.9	31.7	0.8	1.9	42.5	$-$8.9	$-$17.4	51.2
LI$_u$^¶^	$-$0.8	$-$5.3	14.8	$-$1.0	$-$4.7	21.6	1.9	6.9	27.8	1.9	5.0	37.0	1.0	5.0	20.3	0.9	3.2	29.4	$-$1.8	$-$4.9	36.7	$-$3.2	$-$6.8	46.7
LI$_f$(1)^¶^	$-$2.0	$-$13.1	14.9	$-$2.6	$-$11.8	21.9	$-$3.5	$-$12.3	28.5	13.9	37.5	37.0	3.1	15.2	20.3	2.9	9.8	29.6	2.1	5.8	37.0	$-$7.2	$-$15.5	46.7
LI$_f$(2)^¶^	$-$4.6	$-$29.7	15.4	$-$2.1	$-$9.6	22.4	$-$4.1	$-$12.8	32.2	13.6	36.4	37.4	5.4	26.1	20.6	3.1	10.2	30.4	1.9	4.6	41.1	$-$7.3	$-$15.3	47.6
MI$_u$^¶^	$-$1.0	$-$7.0	14.8	$-$1.8	$-$8.2	21.7	0.7	2.4	28.0	0.3	0.7	37.1	0.9	4.3	20.4	0.9	3.0	29.6	$-$1.8	$-$5.0	37.1	$-$3.2	$-$6.9	46.9
MI$_f$(1)^¶^	$-$2.3	$-$15.2	14.9	$-$1.8	$-$8.3	22.0	$-$1.4	$-$5.0	28.7	16.0	43.1	37.0	3.5	17.0	20.3	3.0	9.9	29.7	2.1	5.6	37.1	$-$7.3	$-$15.5	47.0
MI$_f$(2)^¶^	$-$4.7	$-$30.3	15.3	$-$2.4	$-$10.5	22.4	$-$4.4	$-$13.5	32.2	14.1	37.5	37.5	5.5	26.6	20.6	3.2	10.7	30.3	2.1	5.1	41.3	$-$7.2	$-$15.1	47.7
$^\S$bias, standardized bias, and standard error for CC (complete case): ${\beta}_0 = (99.7,310.9,32.1)$ and ${\beta}_{{\rm sex}} =(-15.9,-38.7,41.1)$.																								
^¶^bias, standardized bias, and standard error for all other methods: ${\beta}_0 = (0.1,0.8,14.8)$ and ${\beta}_{{\rm sex}} =(0.5,2.5,21.2)$.																								

Table J.1 in Appendix J of supplementary material available at *Biostatistics* online shows the double robust property of AIPW$_f$. When the dropout models are misspecified but the imputation models are correctly specified (and vice versa), the biases from AIPW$_f$ are negligible. However, when both the dropout and the mean imputation models are misspecified, the biases are practically significant.

Table 2b and Table J.5 in Appendix J of supplementary material available at *Biostatistics* online, show, for 500 and 1000 subjects, the bias, standardized bias, and the empirical standard error for simulation 2. The biases from LI$_u$ imputation and MI$_u$ are negligible, but the biases from IPW$_{u}$, IPW$_{p}$, IPW$_{f}$, AIPW$_{u}$, AIPW$_{f}$, LI$_f$ imputation, and MI$_f$ are bigger and/or practically significant. This is because, as explained in Section 5.2, Graph 2 fails to satisfy u-MAR, p-MAR, and f-MAR.

With respect to efficiency, the standard errors under the IPW methods are larger than those under MI and LI imputation (Table 2a). Moreover, the standard errors under AIPW$_f$ are equal to or less than equal to those under IPW$_f$ when both dropout and imputation models are correctly specified. The standard errors from IPW$_u$, IPW$_p$, and IPW$_f$ are not very different; the standard errors under IPW$_u$ are slightly smaller than those under the other two IPW methods. We would expect to see a larger difference if the sample size were smaller, because unstable weights are more likely to be obtained when using IPW$_p$ or IPW$_f$ than when using IPW$_u$.

In Appendix E of supplementary material available at *Biostatistics* online, we show an additional simulation (simulation 3) to demonstrate bias from methods whose dropout and/or imputation models do not include $D$ (i.e. IPW$_{u}$, IPW$_{p}$, AIPW$_u$, and MI$_u$) when data are f-MAR. We do not follow the sequential data generating mechanism in simulation 3. Instead, $D$ is generated first, so that it can be included in the models for the outcome and dropout process. Tables J.2 and J.3 in Appendix J of supplementary material available at *Biostatistics* online show the bias, standardized bias, and empirical standard error for 500 subjects for simulation 3. Biases from IPW$_f$, AIPW$_f$, and MI$_f$ are negligible, but biases from IPW$_{u}$, IPW$_{p}$, AIPW$_{u}$, and MI$_{u}$ are practically significant.

## 8. Application

In the OCTO study, for the subjects who had at least one lung function measurement, 22.5% of the outcomes are missing due to monotone dropout and 5.8% of the outcomes are intermittent missing. We ignored the 204 subjects with no data on lung function and forced the missingness pattern to be monotone by ignoring the relatively small number of outcomes observed after a subject’s first missing outcome. Data are then available on 437 subjects; 100 (22.9%) of whom have complete data up to visit 5.

To account for the slightly right skewness in the lung function variable (peak expiratory function), we took its square-root transform. The partly conditional model of interest is

(8.1) \begin{align*} E\left({Y_j}|D\geq j, Z\right) &= \beta_0+\beta_1 j + \beta_2 j^2 + \beta_{{\rm sex}} {{\rm sex}} + \beta_{{{\rm age}}} {{\rm age}}_{{\rm base}} +\beta_{{\rm edu}} {{\rm education}} + \beta_{{{\rm smo}}}{{\rm smoke}} + \nonumber \\&\beta_{{\rm sex}1} j \cdot {{\rm sex}} + \beta_{{{\rm age}}1} j \cdot {{\rm age}}_{{\rm base}} + \beta_{{\rm edu1}}\,j\cdot {{\rm education}} + \beta_{{{\rm smo}}1} j \cdot {{\rm smoke}} \label{eq:applicationeq} \end{align*}

where binary variables ${{\rm sex}}=1$ if subject is female, and ${{\rm smoke}}=1$ if a subject has ever smoked.

The dropout and imputation models at visit $j$ include sex, education, smoking status, past outcomes $\bar{Y}_{j-1}$, and baseline age, Mini-Mental State Exam score, instrumental activities of daily living score, and health prevention score (a measure of the degree to which a subject’s health prevents him from doing things that he likes to do). Mini-Mental State Exam score, instrumental activities of daily living score, and health prevention score can tell us about a subject’s cognitive function, physical ableness, and overall health status, respectively. Exploratory analyses suggest that these auxiliary variables are all associated with both lung function and dropout.

If we assume mortal-cohort dDTIC and independent death hold, then the probability of dying between two visits, given past observed lung function data, missingness history, and covariates such as age$_{{\rm base}}$, does not depend on the past missing lung function data. But if we assume that the data were u-MAR, then probability of dying between two visits given covariates and *all* past lung function data does not depend on the missingness history. Under u-MAR, the probability of dropout at visit $j$ given past outcomes, covariates, and survival up to visit $j$ does not depend on lung function data after visit $j-1$ and $D$. But p-MAR allows this probability to depend on subjects who are alive at future visits, and f-MAR allow this probability to depend on $D$.

As a preliminary step in the data analysis we could test the u-MAR assumption if information on death is available by modeling the association between dropout and $D$. In the OCTO study, we found strong associations between the probability of dropout and $D$ while controlling for past outcomes and covariates. Hence, we deduced that u-MAR was implausible in this example.

The parameters from the dropout models fitted for IPW$_f$ showed that dropout was associated with age$_{{\rm base}}$, lung function at last visit, and the auxiliary variables. The strongest predictor was lung function at the last visit. For example, for those who died between visits 3 and 4, the probabilities of dropout between visits 2 and 3 and between 3 and 4 were higher for subjects who had lower lung function at visits 1 and 2, respectively: the estimated log-odds ratios per unit increase in lung function were respectively $-$0.23 (${\rm p}=0.066$) and $-$0.75 (${\rm p}=0.003$).

Table 3 shows estimates from the complete case analysis (i.e. using only subjects with observed lung function at all five visits), LMM, IEE, IPW, AIPW, and MI. Standard errors were calculated by bootstrapping from the original data. Estimates from the complete case analysis are different from the other methods. Since subjects with better lung function are more likely to be observed than those with poorer lung function, the complete cases are not representative of all subjects who remain in the study at each visit. Hence, the complete case analysis is not valid for partly conditional inference, and we focus on the results from IPW, MI, and AIPW.

**Table 3.** Parameter estimate (standard error) of OCTO data application

	$\hat{\beta}_0$	$\hat{\beta}_1$	$\hat{\beta}_2$	$\hat{\beta}_{{\rm sex}}$	$\hat{\beta}_{{{\rm age}}}$	$\hat{\beta}_{{\rm edu}}$	$\hat{\beta}_{{{\rm smo}}}$	$\hat{\beta}_{{\rm sex}1}$	$\hat{\beta}_{{{\rm age}}1}$	$\hat{\beta}_{{\rm edu}1}$	$\hat{\beta}_{{{\rm smo}}1}$
CC	20.326 (0.831)$^{\dagger}$	0.143 (0.108)	$-$0.042 (0.009)$^{\dagger}$	$-$2.502 (0.612)$^{\dagger}$	$-$0.108 (0.086)	0.093 (0.085)	$-$0.135 (0.556)	$-$0.031 (0.070)	0.008 (0.014)	$-$0.004 (0.013)	$-$0.132 (0.071)
LMM	19.612 (0.446)$^{\dagger}$	0.203 (0.093)$^{\dagger}$	$-$0.050 (0.007)$^{\dagger}$	$-$3.084 (0.310)$^{\dagger}$	$-$0.233 (0.049)$^{\dagger}$	0.235 (0.055)$^{\dagger}$	$-$0.873 (0.309)$^{\dagger}$	0.002 (0.055)	0.012 (0.010)	$-$0.007 (0.010)	$-$0.134 (0.057)$^{\dagger}$
IEE	19.657 (0.414)$^{\dagger}$	0.333 (0.111)$^{\dagger}$	$-$0.053 (0.009)$^{\dagger}$	$-$3.085 (0.291)$^{\dagger}$	$-$0.238 (0.052)$^{\dagger}$	0.228 (0.043)$^{\dagger}$	$-$0.872 (0.291)$^{\dagger}$	0.028 (0.070)	0.022 (0.013)	$-$0.022 (0.015)	$-$0.067 (0.069)
IPW$_u$	19.675 (0.438)$^{\dagger}$	0.076 (0.149)	$-$0.038 (0.013)$^{\dagger}$	$-$3.018 (0.315)$^{\dagger}$	$-$0.271 (0.064)$^{\dagger}$	0.249 (0.052)$^{\dagger}$	$-$0.932 (0.324)$^{\dagger}$	0.071 (0.085)	0.026 (0.015)	$-$0.014 (0.019)	$-$0.111 (0.088)
IPW$_p$	19.618 (0.436)$^{\dagger}$	0.156 (0.151)	$-$0.043 (0.011)$^{\dagger}$	$-$2.996 (0.308)$^{\dagger}$	$-$0.272 (0.060)$^{\dagger}$	0.250 (0.050)$^{\dagger}$	$-$0.866 (0.315)$^{\dagger}$	0.041 (0.089)	0.030 (0.016)	$-$0.019 (0.018)	$-$0.120 (0.093)
IPW$_f$	19.806 (0.442)$^{\dagger}$	0.091 (0.147)	$-$0.031 (0.012)$^{\dagger}$	$-$3.073 (0.308)$^{\dagger}$	$-$0.335 (0.078)$^{\dagger}$	0.251 (0.048)$^{\dagger}$	$-$0.772 (0.311)$^{\dagger}$	0.042 (0.084)	0.037 (0.015)$^{\dagger}$	$-$0.024 (0.018)	$-$0.123 (0.088)
AIPW$_u$	19.560 (0.434)$^{\dagger}$	0.218 (0.142)	$-$0.048 (0.013)$^{\dagger}$	$-$2.980 (0.297)$^{\dagger}$	$-$0.246 (0.057)$^{\dagger}$	0.236 (0.049)$^{\dagger}$	$-$0.844 (0.298)$^{\dagger}$	$-$0.032 (0.087)	0.019 (0.022)	$-$0.004 (0.016)	$-$0.112 (0.087)
AIPW$_f$	19.745 (0.460)$^{\dagger}$	0.065 (0.157)	$-$0.034 (0.016)$^{\dagger}$	$-$3.026 (0.314)$^{\dagger}$	$-$0.336 (0.091)$^{\dagger}$	0.256 (0.049)$^{\dagger}$	$-$0.817 (0.312)$^{\dagger}$	0.017 (0.079)	0.036 (0.020)	$-$0.012 (0.014)	$-$0.110 (0.081)
MI$_u$	19.722 (0.420)$^{\dagger}$	0.172 (0.119)	$-$0.045 (0.011)$^{\dagger}$	$-$ (0.293)$^{\dagger}$	0.000 (0.066)	0.014 (0.014)	$-$0.008 (0.013)	$-$0.084 (0.066)			
MI$_f$	19.750 (0.423)$^{\dagger}$	0.079 (0.128)	$-$0.035 (0.012)$^{\dagger}$	$-$3.055 (0.301)$^{\dagger}$	$-$0.253 (0.053)$^{\dagger}$	0.231 (0.047)$^{\dagger}$	$-$0.963 (0.299)$^{\dagger}$	0.003 (0.064)	0.016 (0.013)	$-$0.007 (0.013)	$-$0.086 (0.066)

$^{\dagger}$Denote parameters that are statistically significant at the 5% level. CC, complete case analysis.

In general, the rate of decline in lung function increases over time (MI$_f$: $\hat{\beta_2}$=$-$0.035, ${\rm p}=0.004$), and this rate of decline is also estimated to be greater if we assume mortal-cohort dDTIC and independent death (MI$_u$: $\hat{\beta_2}$=$-$0.045, ${\rm p}=0.000$). This is reflected in Figure 1 (b) and (c). The estimated rate of decline is also different between IPW$_f$ and IPW$_p$: IPW$_p$ suggests a steeper rate of decline than IPW$_f$ (IPW$_p$: $\hat{\beta_2}$ = $-$0.043 , ${\rm p}= 0$; IPW$_f$: $\hat{\beta_2}$ =$-$0.031 , p = 0.013).

IPW$_p$, IPW$_f$, and AIPW$_f$ suggest the rate of decline in lung function among subjects who are older at recruitment is not as steep as that of younger subjects (IPW$_p$: $\hat{\beta}_{{{\rm age}}1}$ = 0.03, p = 0.057; IPW$_f$: $\hat{\beta}_{{{\rm age}}1}$ = 0.037, p = 0.017; AIPW$_f$: $\hat{\beta}_{{{\rm age}}1}$ = 0.036, p=0.07), but this effect is smaller when determined by MI$_u$ and MI$_f$ (MI$_u$: $\hat{\beta}_{{{\rm age}}1}$ = 0.014, p = 0.301; MI$_f$: $\hat{\beta}_{{{\rm age}}1}$ = 0.016, p=0.208).

Overall conclusions about the effects of baseline covariates are, in general, consistent across the missing data methods (IPW, MI, and AIPW), which is reassuring. Using AIPW$_f$, we conclude that holding all other variables constant, (i) smokers have poorer lung function than non-smokers ($\hat{\beta}_{{{\rm smo}}}$ =$-$0.816, p=0.009), (ii) the older a person is at recruitment, the poorer their initial lung function is ($\hat{\beta}_{{{\rm age}}}$=$-$0.336, ${\rm p}=0.000$); MI$_u$ suggests a smaller effect than the other methods ($\hat{\beta}_{{{\rm age}}}$=$-$0.236, ${\rm p}=0.000$), (iii) females have poorer baseline lung function than males ($\hat{\beta}_{sex}$=$-$3.03, ${\rm p}=0.000$), and (iv) the more education a person has, the better their initial lung function is ($\hat{\beta}_{edu}$=0.256, ${\rm p}=0.000$).

## 9. Discussion

We described and compared the assumptions of IPW, LI imputation, MI, and AIPW for making partly conditional inference. IPW$_u$ require mortal-cohort dDTIC and missingness-independent death to hold. MI$_u$ and LI$_u$ imputation require mortal-cohort dDTIC and independent death to hold. AIPW$_u$ requires either (i) u-MAR or (ii) f-MAR, p-MAR and independent death to hold. IPW$_f$, LI$_f$ imputation, MI$_f$ and AIPW$_f$ require f-MAR to hold.

For data sets with a non-negligible number of deaths, the most appropriate method to handle dropout should be chosen on a case-by-case basis. As a guideline, IPW and AIPW may not be appropriate if the survival process depends on the dropout process, and LI$_u$ imputation or MI$_u$ may not be appropriate if the survival process depends on the past missing longitudinal outcomes. Contrary to intuition, using imputation models that condition on D can sometimes induce bias that would not have been present if $D$ were not conditioned on. This is because, as explained in Section 5.2 and demonstrated in simulation 2, imputation methods that condition on $D$ are valid when f-MAR is satisfied, but may be biased otherwise, whereas imputation methods that do not condition on $D$ require other assumptions and so may still be valid. Hence, conditioning on time of death in the imputation model should not be done automatically. We suggest that it may be sensible to apply various methods to a data set as a simple form of sensitivity analysis to see if the conclusions change from one method to another.

When there is dropout and death, Shardell *and others* (2015) provided an AIPW estimator to measure the causal effect of a time-varying exposure on a longitudinal outcome. The current paper provides AIPW estimating equations to obtain partly conditional means of longitudinal outcomes, and to study (possibly non-causal) associations between the outcomes and covariates among survivors. Our AIPW estimators are double robust to model misspecification. Moreover, when the dropout and imputation models are both correctly specified and the underlying assumptions are met, AIPW is at least as efficient as IPW.

IPW$_u$ is a stronger assumption than both IPW$_p$ and IPW$_f$, but IPW$_u$ may be more efficient when u-MAR is true than IPW$_p$ and IPW$_f$, which rely on p-MAR and f-MAR. We could assess the u-MAR assumption if information on death is available by modeling the association between dropout and $D$. Moreover, if we know certain auxiliary variables might make u-MAR more plausible, these variables should be included in the model for dropout.

When the missing outcomes do not follow a multivariate normal distribution, the full conditional specification (van Buuren and Groothuis-Oudshoorn, 2011) is an alternative to the joint multivariate normal MI. Multivariate normal MI and full conditional specification, however, are equivalent for monotone missing continuous outcomes (Seaman and Hughes, 2016). Note that IPW methods can easily handle both continuous and non-continuous outcomes.

If possible, it is better to stratify on $D$ rather than to include $D$ as a covariate in the dropout or imputation model. This is because stratifying on $D$ gives a richer dropout or imputation model that is less likely to be misspecified than just including $D$ as a covariate. But stratification should be avoided if the sample sizes are small in some strata, since we might obtain unstable weights for IPW or obtain very imprecise estimates of the parameters of the imputation models.

In this article, we have focussed on partly conditional estimation. Interpreting partly conditional estimands, unlike unconditional estimands, does not require defining post-death outcomes, e.g. (non-zero) lung functions in dead people. Dufouil *and others* (2004) argued for partly conditional models, saying that “immortal-cohort inference is generally inappropriate unless the longitudinal and survival processes are independent.” Note, however, this view is not held universally: Aalen and Gunnes (2010) argued that an unconditional model provides “a more fair comparison of treatments” when a treatment keeps subjects with poorer outcomes alive for longer. An alternative estimand when comparing two treatments is the survivor average causal effect. This is the effect of treatment on outcome in a group of subjects who would have survived regardless of treatment status. See, e.g. Hayden *and others* (2005), Egleston *and others* (2006), Yang and Small (2016), and references within for estimating this.

Finally, it is important to note that all of the methods in this article rely on the assumption that, conditional on survival at least to the current visit, the probability of observing an outcome at the current visit depends only on the past outcomes and not on the current outcome. However, we cannot rule out the possibility that dropout depends on the current outcome. In the future we will look at ways to assess the sensitivity of the results to a range of assumptions about the dependence of dropout on the current outcome.

## 10. Software

Software in the form of R code, together with a sample input data set and complete documentation is available on request from the corresponding author (lan.wen@mrc-bsu.cam.ac.uk).

## Supplementary Material

Supplementary Data
