# Erratum: Methods for handling longitudinal outcome processes truncated by dropout and death

**DOI:** 10.1093/biostatistics/kxy001

**Published:** 2018-02-15

**Authors:** Lan Wen, Graciela Muniz Terrera, Shaun R Seaman

**Affiliations:** 1 MRC Biostatistics Unit, University of Cambridge, IPH Forvie Site, Robinson Way, Cambridge, UK; 2 Centre for Dementia Prevention, University of Edinburgh, Kennedy Tower, UK

*Biostatistics* (2018) 19, 4, *pp*. 407–425

doi:10.1093/biostatistics/kxx045

The publisher regrets that the information in the second last row of the final three columns of table 3 was incomplete in the originally published version. The table has now been corrected.

