# Supplementary material for: Methods for handling longitudinal outcome processes truncated by dropout and death
Source: Biostatistics. 2017 Sep 26;19(4):407–25. doi: 10.1093/biostatistics/kxx045 (PMC5971107; doi:10.1093/biostatistics/kxx045)
Supplement: Supplementary Data [file kxx045_supplementary_material.pdf]

Biostatistics (2017), **0**, 0, *pp.* 1–21
doi:10.1093/biostatistics/supplementary_material

Supplemental Materials to Methods for handling longitudinal outcome processes truncated by dropout and death

LAN WEN*

MRC Biostatistics Unit, IPH Forvie Site, Robinson Way, Cambridge CB2 0SR, UK and University of Cambridge

lan.wen@mrc-bsu.cam.ac.uk

GRACIELA MUNIZ TERRERA

Centre for Dementia Prevention, University of Edinburgh Kennedy Tower, EH10 5HF, UK

SHAUN R. SEAMAN

MRC Biostatistics Unit, IPH Forvie Site, Robinson Way, Cambridge CB2 0SR, UK and University of Cambridge

APPENDIX

A. Details about LI

In the LI method originally proposed by Diggle *and others* (2007), the change of the underlying outcome is decomposed into a compensator and a martingale increment. They assumed equation (3.1) and $E(\epsilon_j|\bar{Y}_{j-1}, X) = 0$, but with D replaced by J (death was not considered in their work) and with $\phi_j = (\mathbf{0}_{j-2}^T, 1)^T$, where $\mathbf{0}_{j-2}$ denotes a vector of $j-2$ zeroes. They allowed Y_j to be observed with a measurement error that is independent of X and the underlying outcome

© The Author 2017. Published by Oxford University Press. All rights reserved. For permissions, please e-mail: journals.permissions@oup.com

process. Seaman *and others* (2016), building on the work of Aalen and Gunnes (2010), discussed how $E(Y_j - Y_{j-1}|\bar{Y}_{j-1}, X)$ can be allowed to depend on $\bar{Y}_{j-1}$ and discussed partly-conditional inference. When data are monotone missing, the resulting model is that of expressions (3.1) and (3.6). Seaman *and others* (2016) proposed several methods for estimating the parameters in this model. They assumed that when data are monotone missing and $E(Y_j - Y_{j-1}|\bar{Y}_{j-1}, X)$ is allowed to depend on all of $\bar{Y}_{j-1} = (Y_1, \ldots, Y_{j-1})$, these methods are all equivalent to fitting the model of expressions (3.1) and (3.2) by maximum likelihood (ML) and imputing missing outcomes Y_j sequentially as $\rho_j + \phi_j^T \bar{Y}_{j-1} + \psi_j^T X$, setting ρ_j, ϕ_j, and ψ_j to equal to their ML estimates. This procedure is the same as MI, except that only one data set is imputed, ρ_j, ϕ_j, and ψ_j are set to equal to their ML estimates rather than drawn from a posterior distribution, and no error ϵ_j is added to the imputed values. As with MI, post-death imputed outcomes should be deleted before analysing the imputed data set. Variances are estimated by bootstrap. The LI imputation method provides consistent estimation of the parameters of a model for $E(Y_j|Z, D \geqslant j)$ provided that expressions (3.1) and (3.6), and mortal-cohort dDTIC and independent death hold (Seaman *and others*, 2016).

B. Example of mortal cohort dDTIC and MAR

As we note, mortal-cohort dDTIC is the assumption that $P(\bar{R}_j = \bar{r}_j \mid \bar{Y}_j, X, D \geqslant j) = P(\bar{R}_j = \bar{r}_j \mid \bar{Y}_{j-1}, X, D \geqslant j)$ for all j and all $\bar{r}_j$. MAR conditional on subjects being alive would be the assumption that $P(\bar{R}_j = \bar{r}_j \mid \bar{Y}_j, X, D \geqslant j) = P(\bar{R}_j = \bar{r}_j \mid \bar{Y}_{j,\text{obs}}(\bar{r}_j), X, D \geqslant j)$ for all j, where $\bar{Y}_{j,\text{obs}}(\bar{r}_j)$ denotes the elements of $\bar{Y}_j$ that are observed when $\bar{R}_j = \bar{r}_j$. The following simple example shows that mortal cohort dDTIC does not imply MAR conditional on subjects being alive.

Let $J = 3$ and $P(Y_2 = Y_3 = 0) = 1$. Suppose that only the five values of (Y_2, R_2, R_3, D) in the following table have non-zero probability. The table shows the probabilities of these values.

Y_2	R_2	R_3	D	Probability
0	0	0	3	1/8
0	1	1	3	1/4
0	0	0	2	1/8
1	1	1	3	1/4
1	0	0	2	1/4

It can be seen that $P(R_2 = R_3 = 0 \mid Y_2, D \geqslant 3)$ equals $1/3$ if $Y_2 = 0$ and equals 0 if $Y_2 = 1$. As this depends on the unobserved Y_2, the data are not MAR conditional on subjects being alive. However, since $P(R_2 = R_3 = 0 \mid Y_2, Y_3, D \geqslant 3) = P(R_2 = R_3 = 0 \mid Y_2, D \geqslant 3)$ and $P(R_2 = 0 \mid Y_2, D \geqslant 2) = P(R_2 = 0 \mid D \geqslant 2) = 1/2$, the data do satisfy mortal-cohort dDTIC.

C. Details on "Most complex scenarios for mortal-cohort dDTIC and independent death to hold"

Here we show that Graph 2 does not satisfy f-MAR. We use d-separation as a procedure to determine conditional independence, and we show that R_2 is not conditionally independent of Y_2 given Y_1, D and X. An analogous argument shows that, more generally, R_k is not independent of Y_k given R_{k-1}, $\bar{Y}_{k-1}$, D and X.

We let $J = 3$ (the argument is the same when $J > 3$). Conditioning on D is the same as conditioning on the set of indicator variables $\{I(D \geqslant 2), I(D \geqslant 3)\}$. After dropping all of the variables that are neither included in the set $\{R_2, X, Y_1, Y_2, I(D \geqslant 2), I(D \geqslant 3)\}$ nor are ancestors of the variables in that set, we are left with directed acyclic graphs displayed in Figure C.1.

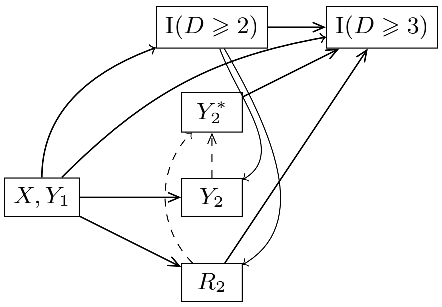

Figure C.1: Directed acyclic graph for $J = 3$ after removing variables not in the set $\{R_2, Y_1, Y_2, I(D \geqslant 2), I(D \geqslant 3)\}$ nor in the set of their ancestors

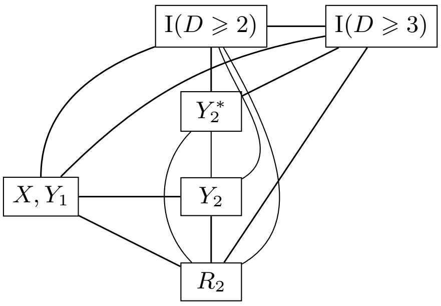

Figure C.2: Graph for $J = 4$ after moralizing Graph C.1

After moralizing the graph, we are left with the conditional independence graph displayed in Figure C.2. It can be seen that there is a path, $Y_2 - R_2$, from Y_2 to R_2 that is not blocked by the set $\{Y_1, X, I(D \geqslant 2), I(D \geqslant 3)\}$. Hence, Y_2 is not independent of R_2 given Y_1, X, and D.

The argument to show that Graph 2 does not satisfy p-MAR is almost identical. We do this by showing that R_2 is not independent of Y_2 given Y_1, X and $I(D \geqslant 3)$. Since $I(D \geqslant 2)$ is a parent of $I(D \geqslant 3)$ in Graph 2, after dropping all the variables that are neither included in the set $\{R_2, Y_1, Y_2, I(D \geqslant 2), I(D \geqslant 3)\}$ no are ancestors of the variables in this set, we are again left with the directed acyclic graph given in C.1. The resulting conditional independence graph is therefore, again, that given in Figure C.2. It can be seen that there is a path, $R_2 - Y_2$, from Y_2 to R_2 that is not blocked by the set $\{Y_1, X, I(D \geqslant 3)\}$.

D. Details of simulations 1 and 2

In order to calculate the true values of parameters β_k in the analysis model, we simulated data for $N = 10^9$ individuals and calculated the average outcome at each visit for males and females separately.

In simulations 1 and 2, the baseline covariate is generated and the parameters for the longitudinal, survival and dropout models are chosen to mimic the data from the OCTO study. Data are generated in a sequential manner, and we assume a simplified version of Directed Acyclic

Graphs (DAGs) 1 and 2, respectively (Figures D.1a and D.1b). Note that in Figure D.1a, Y_j, R_j, and $I(D \geqslant j)$ depend on Y_{j-1}, but not on $\bar{Y}_{j-2}$. In Figure D.1b, Y_j and R_j depend on Y_{j-1}, but not on $\bar{Y}_{j-2}$; $I(D \geqslant j)$ depends on the last *observed* outcome, but not on the ones before.

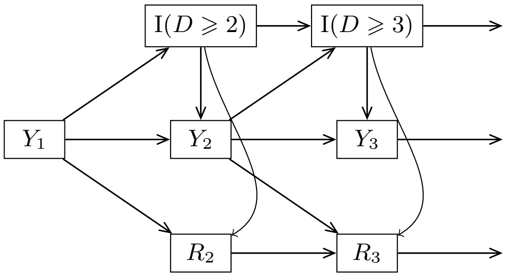

(a) Simplified DAG 1 for Simulation 1

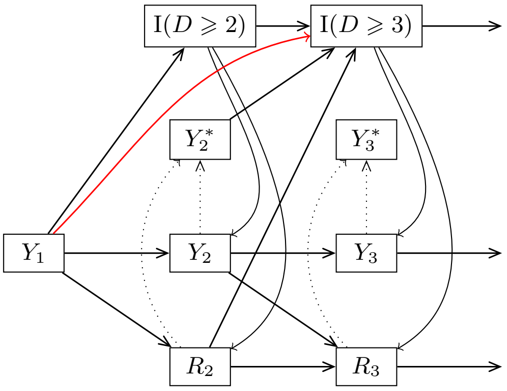

(b) Simplified DAG 2 for Simulation 2. If $Y_2^* \neq \varnothing$, then omit the dependence in red.

Figure D.1

D.1 *Simulation 1 under scenario 1*

In simulation 1, data is generated according to DAG D.1a. The longitudinal outcome, survival and dropout times are generated using the models below. Assume $Y_1 \sim N(17.63, 2.349^2)$. For $j = \{2, 3, 4, 5\}$, while $D \geqslant j$, let:

$$Y_j = \beta^s_{0,j} + \beta^s_{1,j} Y_{j-1} + \beta^s_{2,j}\text{sex} + \epsilon_j \tag{D.1}$$

j=2:	$\beta^s_{0,2}$=5.295,	$\beta^s_{1,2}$ =0.7349,	$\beta^s_{2,2}$ =-0.5150,	$\epsilon_2 \sim N(0, 1.850^2)$
j=3:	$\beta^s_{0,3}$=6.537,	$\beta^s_{1,3}$ =0.6445,	$\beta^s_{2,3}$ =-0.7152,	$\epsilon_3 \sim N(0, 1.818^2)$
j=4:	$\beta^s_{0,4}$=4.181,	$\beta^s_{1,4}$ =0.7420,	$\beta^s_{2,4}$ =-0.7740,	$\epsilon_4 \sim N(0, 1.787^2)$
j=5:	$\beta^s_{0,5}$=4.201,	$\beta^s_{1,5}$ =0.7165 ,	$\beta^s_{2,5}$ =-0.0203,	$\epsilon_5 \sim N(0, 1.971^2)$

Survival model at visit j is

$$\begin{aligned}\text{logit} P(D \geqslant j | D \geqslant j-1, \bar{Y}_{j-1}, \bar{R}_{j-1}, X) &= \text{logit} P(D \geqslant j | D \geqslant j-1, Y_{j-1}, X) \\ &= \delta_{0,j} + \delta_{1,j} Y_{j-1} + \delta_{2,j}\text{sex} \end{aligned} \tag{D.2}$$

j=2:	$\delta_{0,2}$ =-1.127, $\delta_{1,2}$ =0.1935, $\delta_{2,2}$ =0.3505
j=3:	$\delta_{0,3}$ =-5.909, $\delta_{1,3}$ =0.4323, $\delta_{2,3}$ = 0.4040
j=4:	$\delta_{0,4}$ =-3.200, $\delta_{1,4}$ =0.2418, $\delta_{2,4}$ = 0.9848
j=5:	$\delta_{0,5}$ =-3.210, $\delta_{1,5}$ =0.2519, $\delta_{2,5}$ = 0.6246

dropout model, for all $j = \{2, 3, 4, 5\}$,

$$\begin{aligned}\text{logit}P(R_j = 1|R_{j-1} = 1, X, \bar{Y}_j, D \geqslant j) =& \text{logit}P(R_j = 1|R_{j-1} = 1, X, Y_{j-1}, D \geqslant j) \\ =& \omega_{0,j} + \omega_{1,j}Y_{j-1} + \omega_{2,j}\text{sex} \qquad \text{(D.3)}\end{aligned}$$

j=2:	$\omega_{0,2}$ =-2.109, $\omega_{1,2}$ =0.1931, $\omega_{2,2}$ = -0.0862
j=3:	$\omega_{0,3}$ =-3.593, $\omega_{1,3}$ =0.2421, $\omega_{2,3}$ =0.6357
j=4:	$\omega_{0,4}$ =-1.959, $\omega_{1,4}$ =0.1756, $\omega_{2,4}$ =0.7582
j=5:	$\omega_{0,5}$ =-3.432, $\omega_{1,5}$ =0.2507, $\omega_{2,5}$ =0.5435

D.2 *Simulation 2 under scenario 2*

In simulation 2, data is generated according to DAG D.1b. We define A_j to indicate the last observed visit before j. For example, if $\bar{R}_3 = \{1, 1, 0\}$, then $A_3 = 2$. The longitudinal and dropout models and their corresponding parameters are the same as those in equations (D.1) and (D.3). The survival model for j and $A_{j-1} = a_{j-1}$ is

$$\begin{aligned}\text{logit}P(D \geqslant j|D \geqslant j-1, \bar{Y}_{j-1}, X) &= \text{logit}P(D \geqslant j|D \geqslant j-1, Y_{A_{j-1}}, X) \\ &= \delta_{0,j} + \delta_{1,j}Y_{A_{j-1}} + \delta_{2,j}\text{sex}\end{aligned}$$

j=2	$A_1 = 1$:	$\delta_{0,2}$ = -1.127, $\delta_{1,2}$ = 0.1935, $\delta_{2,2}$ = 0.3505
j=3	$A_2 = 2$:	$\delta_{0,3}$ =-5.561, $\delta_{1,3}$ =0.4323, $\delta_{2,3}$ = 0.4040
j=3	$A_2 = 1$:	$\delta_{0,3}$ =2.429, $\delta_{1,3}$ =-0.0979, $\delta_{2,3}$ = 1.024
j=4	$A_3 = 3$:	$\delta_{0,4}$ =-2.695, $\delta_{1,4}$ =0.2418, $\delta_{2,4}$ = 0.9848
j=4	$A_3 = 2$:	$\delta_{0,4}$ =5.540, $\delta_{1,4}$ =0.4973, $\delta_{2,4}$ = 1.570
j=4	$A_3 = 1$:	$\delta_{0,4}$ =0.5887, $\delta_{1,4}$ =0.0554, $\delta_{2,4}$ = 0.4973
j=5	$A_4 = 4$:	$\delta_{0,5}$ =-2.446, $\delta_{1,5}$ =0.2519, $\delta_{2,5}$ = 0.6246
j=5	$A_4 = 3$:	$\delta_{0,5}$ =-1.441, $\delta_{1,5}$ =-0.0657, $\delta_{2,5}$ = 3.228
j=5	$A_4 = 2$:	$\delta_{0,5}$ =3.208, $\delta_{1,5}$ =-0.2185, $\delta_{2,5}$ = 0.0141
j=5	$A_4 = 1$:	$\delta_{0,5}$ =-8.625, $\delta_{1,5}$ =1.089, $\delta_{2,5}$ = 1.527

E. Simulation 3

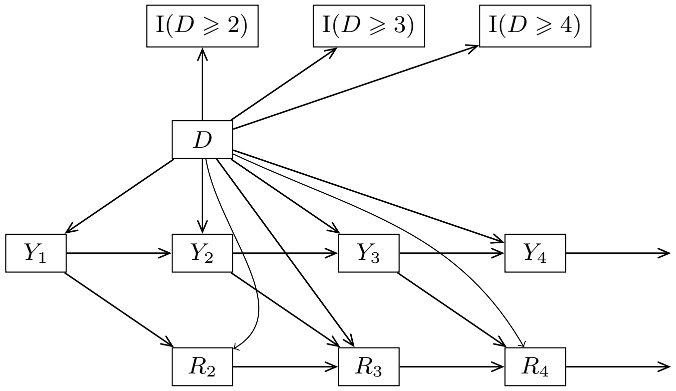

Figure E.1: DAG 3 of simulation 3

Here we show the details of simulation 3 under Directed Acyclic Graph 3 (figure E.1) for $N = 500$. In this simulation, D is generated first. Then the longitudinal outcomes, and dropout times are generated afterwards. Both of the longitudinal and dropout models depend on D, and missing outcomes due to dropout are removed afterwards. Note that we can also model the time of death and longitudinal outcomes in a shared-parameter joint model.

For simplicity, we assume the following analysis model:

$$E(Y_j|D \geqslant j) = \beta_0 + \beta_1 \mathrm{I}(j=2) + \beta_2 \mathrm{I}(j=3) + \beta_3 \mathrm{I}(j=4) + \beta_4 \mathrm{I}(j=5) \tag{E.1}$$

We assume that time of death follows $\mathrm{N}(7.492, 3.714^2)$ distribution. The longitudinal outcomes and the dropout times are generated using the models below. For $j = \{2,3,4,5\}$, while $D \geqslant j$, let:

$$Y_j = \beta^s_{0,j} + \beta^s_{1,j} Y_{j-1} + \beta^s_{2,j}\mathrm{sex} + \beta^s_{3,j} D + \epsilon_j \tag{E.2}$$

j=2:	$\beta^s_{0,2}$=5.054, $\beta^s_{1,2}$ =0.7316, $\beta^s_{2,2}$ =-0.5662, $\beta^s_{3,2}$ =0.0520, $\epsilon_2 \sim N(0, 1.850^2)$
j=3:	$\beta^s_{0,3}$=5.162, $\beta^s_{1,3}$ =0.6456, $\beta^s_{2,3}$ =-0.8348, $\beta^s_{3,3}$ =0.1980, $\epsilon_3 \sim N(0, 1.804^2)$
j=4:	$\beta^s_{0,4}$=3.959, $\beta^s_{1,4}$ =0.7414, $\beta^s_{2,4}$ =-0.7798, $\beta^s_{3,4}$ =0.0309, $\epsilon_4 \sim N(0, 1.793^2)$
j=5:	$\beta^s_{0,5}$=4.201, $\beta^s_{1,5}$ =0.7165, $\beta^s_{2,5}$ =-0.0203,$\epsilon_5 \sim N(0, 1.971^2)$

dropout model, for all $j = \{2, 3, 4, 5\}$, with D in X, is

$$\begin{aligned}\text{logit}P(R_j = 1|R_{j-1} = 1, X, \bar{Y}_D, D \geqslant j) =& \text{logit}P(R_j = 1|R_{j-1} = 1, X, Y_{j-1}, D \geqslant j) \\ =& \omega_{0,j} + \omega_{1,j}Y_{j-1} + \omega_{2,j}\text{sex} + \omega_{3,j}\text{D} \end{aligned} \tag{E.3}$$

j=2:	$\omega_{0,2}$ =-3.100, $\omega_{1,2}$ =0.1801, $\omega_{2,2}$ =-0.3588, $\omega_{3,2}$ = 0.2403
j=3:	$\omega_{0,3}$ =-6.968, $\omega_{1,3}$ =0.2518, $\omega_{2,3}$ =0.1700, $\omega_{3,3}$ = 0.5238
j=4:	$\omega_{0,4}$ =-6.459, $\omega_{1,4}$ =0.1442, $\omega_{2,4}$ =0.3834, $\omega_{3,4}$ = 0.7136
j=5:	$\omega_{0,5}$ =-3.432, $\omega_{1,5}$ =0.2507, $\omega_{2,5}$ =0.5435

F. Proof that mortal-cohort dDTIC $f(Y_j|\bar{Y}_{j-1}, \bar{R}_j, X, D \geqslant j) = f(Y_j|\bar{Y}_{j-1}, X, D \geqslant j)$ is equivalent to $f(\bar{R}_j|\bar{Y}_j, X, D \geqslant j) = f(\bar{R}_j|\bar{Y}_{j-1}, X, D \geqslant j)$

$$\begin{aligned} & f(Y_j|\bar{Y}_{j-1}, \bar{R}_j, X, D \geqslant j) = f(Y_j|\bar{Y}_{j-1}, X, D \geqslant j) \\ \iff & \frac{f(\bar{Y}_j, \bar{R}_j, X, D \geqslant j)}{f(\bar{Y}_{j-1}, \bar{R}_j, X, D \geqslant j)} = \frac{f(\bar{Y}_j, X, D \geqslant j)}{f(\bar{Y}_{j-1}, X, D \geqslant j)} \\ \iff & \frac{f(\bar{R}_j|\bar{Y}_j, X, D \geqslant j) f(\bar{Y}_j, X, D \geqslant j)}{f(\bar{R}_j|\bar{Y}_{j-1}, X, D \geqslant j) f(\bar{Y}_{j-1}, X, D \geqslant j)} = \frac{f(\bar{Y}_j, X, D \geqslant j)}{f(\bar{Y}_{j-1}, X, D \geqslant j)} \\ \therefore\ & f(\bar{R}_j|\bar{Y}_j, X, D \geqslant j) = f(\bar{R}_j|\bar{Y}_{j-1}, X, D \geqslant j) \end{aligned}$$

G. Proof of Theorem 1

Theorem 1: u-MAR holds if and only if mortal-cohort dDTIC and missingness-independent death hold.

We start by proving (1) mortal-cohort dDTIC and missingness-independent death $\implies$ u-MAR, then we prove (2) u-MAR $\implies$ mortal-cohort dDTIC and missingness-independent death.

(1) Mortal-cohort dDTIC and missingness-independent death $\implies$ u-MAR

To prove (1), *first*, we prove by induction that mortal-cohort dDTIC can also be written as

$$P(R_{k+1} = 1|R_k = 1, \bar{Y}_j, D \geqslant j, X) = P(R_{k+1} = 1|R_k = 1, \bar{Y}_{j-1}, D \geqslant j, X), \quad \forall k + 1 \leqslant j$$

Base case: For any j, suppose that only the first visit was observed such that $\bar{R}_j = (1, 0, 0, \ldots, 0)^T$,

then by the mortal-cohort dDTIC

$$P(R_1 = 1, R_2 = \ldots = R_j = 0|\bar{Y}_j, D \geqslant j, X) = P(R_1 = 1, R_2 = \ldots = R_j = 0|\bar{Y}_{j-1}, D \geqslant j, X)$$

Under a monotone missing pattern, this implies

$$P(R_2 = 1|R_1 = 1, \bar{Y}_j, D \geqslant j, X) = P(R_2 = 1|R_1 = 1, \bar{Y}_{j-1}, D \geqslant j, X)$$

Induction assumption: Suppose that $P(R_p = 1|R_{p-1} = 1, \bar{Y}_j, D \geqslant j, X) = P(R_p = 1|R_{p-1} = 1, \bar{Y}_{j-1}, D \geqslant j, X)$, for all $p \leqslant k < j$ (i.e. we observe all visits up to and including visit p). We shall show that

$$P(R_{k+1} = 1|R_k = 1, \bar{Y}_j, D \geqslant j, X) = P(R_{k+1} = 1|R_k = 1, \bar{Y}_{j-1}, D \geqslant j, X), \forall k + 1 \leqslant j \quad \text{(G.1)}$$

Suppose that the missingness history up to and including visit j is $\{R_1 = 1, \ldots, R_k = 1, R_{k+1} = 0, \ldots R_j = 0\}$ (i.e. all visits up to and including visit k were observed). The left hand side of $P(\bar{R}_j|\bar{Y}_j, D \geqslant j, X) = P(\bar{R}_j|\bar{Y}_{j-1}, D \geqslant j, X)$ is

$$P(R_{k+1} = 0|R_k = 1, \bar{Y}_j, D \geqslant j, X) \left[\prod_{p=2}^{k} P(R_p = 1|R_{p-1} = 1, \bar{Y}_j, D \geqslant j, X)\right] \left[\prod_{q=k+2}^{j} P(R_q = 0|R_{q-1} = 0, \bar{Y}_j, D \geqslant j, X)\right]$$

and the right hand side of $P(\bar{R}_j|\bar{Y}_j, D \geqslant j, X) = P(\bar{R}_j|\bar{Y}_{j-1}, D \geqslant j, X)$ is

$$P(R_{k+1} = 0|R_k = 1, \bar{Y}_{j-1}, D \geqslant j, X) \left[\prod_{p=2}^{k} P(R_p = 1|R_{p-1} = 1, \bar{Y}_{j-1}, D \geqslant j, X)\right] \times \left[\prod_{q=k+2}^{j} P(R_q = 0|R_{q-1} = 0, \bar{Y}_{j-1}, D \geqslant j, X)\right]$$

Using the induction assumption, equation (G.1) is true. Hence, mortal-cohort dDTIC can be written as

$$P(R_{k+1} = 1|R_k = 1, \bar{Y}_j, D \geqslant j, X) = P(R_{k+1} = 1|R_k = 1, \bar{Y}_{j-1}, D \geqslant j, X), \quad \forall k + 1 \leqslant j$$

Second, we shall show that

$$\text{(i). } P(R_{k+1}=1|R_k=1,\bar{Y}_j,D=j,X)=P(R_{k+1}=1|R_k=1,\bar{Y}_j,D\geqslant j,X)$$
$$=P(R_{k+1}=1|R_k=1,\bar{Y}_{j-1},D\geqslant j,X),\quad \forall k+1\leqslant j,\ j\geqslant 3 \tag{G.2}$$

and

$$\text{(ii). } P(R_{k+1}=1|R_k=1,\bar{Y}_{j-1},D\geqslant j,X)=P(R_{k+1}=1|R_k=1,\bar{Y}_{j-1},D\geqslant j-1,X),\quad \forall k+1\leqslant j,\ j\geqslant 3$$

Equations in (i) hold because,

$$P(R_{k+1}=1|R_k=1,\bar{Y}_j,D=j,X)$$
$$=P(R_{k+1}=1|R_k=1,\bar{Y}_j,D\geqslant j,X)\frac{1-P(D\geqslant j+1|D\geqslant j,\bar{R}_{k+1}=\mathbf{1},\bar{Y}_j,X)}{1-P(D\geqslant j+1|D\geqslant j,\bar{R}_k=\mathbf{1},\bar{Y}_j,X)}$$
$$=P(R_{k+1}=1|R_k=1,\bar{Y}_j,D\geqslant j,X) \tag{G.3}$$
$$=P(R_{k+1}=1|R_k=1,\bar{Y}_{j-1},D\geqslant j,X) \tag{G.4}$$

Equation (G.3) is true because of missingness-independent death, and equation (G.4) is true because of mortal-cohort dDTIC.

Equation (ii) holds because,

$$P(R_{k+1}=1|R_k=1,\bar{Y}_{j-1},D\geqslant j,X)$$
$$=P(R_{k+1}=1|R_k=1,\bar{Y}_{j-1},D\geqslant j-1,X)\frac{P(D\geqslant j|D\geqslant j-1,\bar{R}_{k+1}=\mathbf{1},\bar{Y}_{j-1},X)}{P(D\geqslant j|D\geqslant j-1,\bar{R}_k=\mathbf{1},\bar{Y}_{j-1},X)}$$
$$=P(R_{k+1}=1|R_k=1,\bar{Y}_{j-1},D\geqslant j-1,X) \tag{G.5}$$

Equation (G.5) is true because of missingness-independent death. It can be shown then, by induction (applying mortal-cohort dDTIC and missingness-independent death iteratively),

$$P(R_{k+1}=1|R_k=1,\bar{Y}_{j-1},D\geqslant j,X)=P(R_{k+1}=1|R_k=1,\bar{Y}_k,D\geqslant k+1,X)$$

Thus, by mortal-cohort dDTIC and missingness-independent death, u-MAR holds.

(2) u-MAR $\Longrightarrow$ mortal-cohort dDTIC and missingness-independent death

Note that by Bayes' theorem, it can be shown that the following equations are true under u-MAR:

$$P(R_k=1|R_{k-1}=1,\bar{Y}_j,D=j,X)=P(R_k=1|R_{k-1}=1,\bar{Y}_j,D\geqslant j,X)$$
$$=P(R_k=1|R_{k-1}=1,\bar{Y}_{k-1},D\geqslant k,X),\quad \forall j\geqslant k$$

1. First we note that under monotone missing data, it can be shown that mortal-cohort dDTIC can be written as $P(R_j = 1|D \geqslant j, \bar{Y}_j, X) = P(R_j = 1|D \geqslant j, \bar{Y}_{j-1}, X)$. We shall show that $P(R_j = 1|D \geqslant j, \bar{Y}_j, X) = P(R_j = 1|D \geqslant j, \bar{Y}_{j-1}, X)$ when u-MAR holds.

$$\begin{aligned} P(R_j = 1|D \geqslant j, \bar{Y}_j, X) &= \prod_{k=2}^{j} P(R_k = 1|R_{k-1} = 1, D \geqslant j, \bar{Y}_j, X) \\ &\overset{\text{(u-MAR)}}{=} \prod_{k=2}^{j} P(R_k = 1|R_{k-1} = 1, D \geqslant j, \bar{Y}_{j-1}, X) \\ &= P(R_j = 1|D \geqslant j, \bar{Y}_{j-1}, X) \end{aligned}$$

2. We shall show that $P(R_j = 1|D \geqslant j+1, \bar{Y}_j, X) = P(R_j = 1|D \geqslant j, \bar{Y}_j, X)$ (i.e. missingness-independent death holds) when u-MAR holds.

$$\begin{aligned} P(R_j = 1|D \geqslant j+1, \bar{Y}_j, X) &= \prod_{k=2}^{j} P(R_k = 1|R_{k-1} = 1, D \geqslant j+1, \bar{Y}_j, X) \\ &\overset{\text{(u-MAR)}}{=} \prod_{k=2}^{j} P(R_k = 1|R_{k-1} = 1, D \geqslant j, \bar{Y}_j, X) \\ &= P(R_j = 1|D \geqslant j, \bar{Y}_j, X) \end{aligned}$$

H. Validity of MI$_f$

Let us assume equations (4.12) and (4.13) for $D = l$, and $\bar{Y}_l|X, D = l \sim \mathrm{N}(\mu^{(l)}, \Sigma^{(l)})$. We would like to show that under f-MAR, MI$_f$ will be valid. It can be shown that f-MAR can be written as $P(\bar{R}_k|D = l, \bar{Y}_l, X) = P(\bar{R}_k|D = l, \bar{Y}_{k-1}, X)$,$\forall k \leqslant l$, which is equivalent to

$$f(Y_l, \ldots, Y_k|\bar{R}_k, D = l, \bar{Y}_{k-1}, X) = f(Y_l, \ldots, Y_k|D = l, \bar{Y}_{k-1}, X) \quad \forall k \leqslant l$$

Therefore, if $R_{k-1} = 1, R_k = 0$

$$f(Y_j|R_{k-1} = 1, R_k = 0, \ldots, R_j = 0, D = l, \bar{Y}_{k-1}, X) = f(Y_j|D = l, \bar{Y}_{k-1}, X) \quad \forall k \leqslant j$$

For subjects whose $D = l$, let η_j denote the value of Y_j in the data set created by MI (as $N \to \infty$) with true values of $\mu^{(l)}$ and $\Sigma^{(l)}$.

$$\begin{aligned} \eta_j &= \begin{cases} Y_j, & \text{if } R_j = 1 \\ E(Y_j|\bar{Y}_l, D = l, X), & \text{if } R_j = 0 \end{cases} \\ &= \begin{cases} Y_j, & \text{if } R_j = 1 \\ E(Y_j|\bar{Y}_U, D = l, X), & \text{if } R_j = 0 \end{cases} \end{aligned}$$

where U is the last visit that the outcome is observed. Then,

$$E(\eta_j|D \geqslant j, X) = \sum_{\forall l \geqslant j} \Big\{ E(Y_j|R_j = 1, D \geqslant j, D = l, X) P(R_j = 1|D \geqslant j, D = l, X) P(D = l|D \geqslant j, X) +$$
$$E(\eta_j|R_j = 0, D \geqslant j, D = l, X) P(R_j = 0|D \geqslant j, D = l, X) P(D = l|D \geqslant j, X) \Big\}$$
$$= \sum_{\forall l \geqslant j} \Big\{ E(Y_j|R_j = 1, D \geqslant j, D = l, X) P(R_j = 1|D \geqslant j, D = l, X) P(D = l|D \geqslant j, X) +$$
$$E_{U,\bar{Y}_U}[E(Y_j|\bar{Y}_U, D = l, D \geqslant j, X)|R_j = 0, D \geqslant j, D = l, X] P(R_j = 0|D \geqslant j, D = l, X) P(D = l|D \geqslant j, X) \Big\}$$

Note that conditioning on $\{R_U = 1, R_{U+1} = \ldots = R_j = 0\}$ is equivalent to conditioning on $(U, \{R_j = 0\})$, which is equivalent to conditioning on U. Then, by f-MAR

$$E_{U,\bar{Y}_U}[E(Y_j|\bar{Y}_U, D = l, D \geqslant j, X)|R_j = 0, D \geqslant j, D = l, X]$$
$$= E_U\{E_{\bar{Y}_U}[E(Y_j|\bar{Y}_U, U, D = l, D \geqslant j, X, R_j = 0)|U, R_j = 0, D \geqslant j, D = l, X]|R_j = 0, D \geqslant j, D = l, X\}$$
$$= E(Y_j|R_j = 0, D \geqslant j, D = l, X)$$

Therefore,

$$E(\eta_j|D \geqslant j, X) = \sum_{\forall l \geqslant j} \Big\{ E(Y_j|R_j = 1, D \geqslant j, D = l, X) P(R_j = 1|D \geqslant j, D = l, X) P(D = l|D \geqslant j, X) +$$
$$E(Y_j|R_j = 0, D \geqslant j, D = l, X) P(R_j = 0|D \geqslant j, D = l, X) P(D = l|D \geqslant j, X) \Big\}$$
$$= E(Y_j|D \geqslant j, X)$$

Since the true values of $\mu^{(l)}$ and $\Sigma^{(l)}$ are unknown, we can replace them by their maximum likelihood estimates $\hat{\mu}^{(l)}$ and $\hat{\Sigma}^{(l)}$. If we assume equations (4.12) and (4.13), under f-MAR, it can be shown that $(\hat{\mu}^{(l)}, \hat{\Sigma}^{(l)})$ are consistent parameter estimates of $(\mu^{(l)}, \Sigma^{(l)})$. Since we explained in Section 3.2 that MI_f and LI imputation_f are very similar, we would expect both MI_f and LI imputation_f to be valid.

I. Proof of Theorem 2

We will first show how AIPW estimating equations (equation (6.14)) are derived from AIPWCC in Seaman and Copas (2009). Ignoring any terms in AIPW estimating equations that contains R_j for $j > D$, the AIPW estimating equations can be written as

$$\begin{aligned}\Psi(\beta,\alpha,\gamma) =& \frac{R_J}{\tilde{\pi}_J(\bar{Y}_{J-1},X;\alpha)}U(\beta) + \sum_{j=1}^{J-1}\left(\frac{R_j}{\tilde{\pi}_j(\bar{Y}_{j-1},X;\alpha)} - \frac{R_{j+1}}{\tilde{\pi}_{j+1}(\bar{Y}_j,X;\alpha)}\right)H_j(\bar{Y}_j,X;\beta,\gamma)\\
&= I(D=J)\frac{R_D}{\tilde{\pi}_D(\bar{Y}_{D-1},X;\alpha)}U(\beta) + I(D<J)\frac{R_D}{\tilde{\pi}_D(\bar{Y}_{D-1},X;\alpha)}H_D(\bar{Y}_D,X;\beta,\gamma)+\\
&\sum_{j=1}^{\min(D-1,J-1)}\left(\frac{R_j}{\tilde{\pi}_j(\bar{Y}_{j-1},X;\alpha)} - \frac{R_{j+1}}{\tilde{\pi}_{j+1}(\bar{Y}_j,X;\alpha)}\right)H_j(\bar{Y}_j,X;\beta,\gamma)\\
&= \frac{R_D}{\tilde{\pi}_D(\bar{Y}_{D-1},X;\alpha)}U(\beta) + \sum_{j=1}^{D-1}\left(\frac{R_j}{\tilde{\pi}_j(\bar{Y}_{j-1},X;\alpha)} - \frac{R_{j+1}}{\tilde{\pi}_{j+1}(\bar{Y}_j,X;\alpha)}\right)H_j(\bar{Y}_j,X;\beta,\gamma)\end{aligned}$$

Theorem 2: Under f-MAR, the AIPW$_f$ gives consistent estimations if either the dropout model or the model $H_j(\bar{Y}_j,X;\beta,\gamma)$ is correctly specified.

Under Theorem 2, we assume f-MAR:

$$P(R_j=1|R_{j-1}=1,\bar{Y}_D,X) = P(R_j=1|R_{j-1}=1,\bar{Y}_{j-1},X), \quad \forall j$$

and equivalently written as $f(Y_D,\ldots,Y_j|\bar{Y}_{j-1},R_j=1,X) = f(Y_D,\ldots,Y_j|\bar{Y}_{j-1},R_{j-1}=1,X)$. Equation (6.14) can be written as

$$\Psi(\beta,\alpha,\gamma) = \frac{R_1}{\tilde{\pi}_1(X;\alpha)}H_1(Y_1,X;\beta,\gamma) + \sum_{j=2}^{D}\frac{R_j}{\tilde{\pi}_j(\bar{Y}_{j-1},X;\alpha)}\left[H_j(\bar{Y}_j,X;\beta,\gamma) - H_{j-1}(\bar{Y}_{j-1},X;\beta,\gamma)\right] \quad \text{(I.1)}$$

Let $\hat{\alpha},\hat{\gamma}$ be consistent estimators of α,γ, and let α_o,γ_o be the limiting values of $\hat{\alpha},\hat{\gamma}$. Let $\tilde{\pi}_{oj} = \tilde{\pi}_{oj}(\bar{Y}_{j-1},X) = \tilde{\pi}(\bar{Y}_{j-1},X;\alpha_o)$ and $H_{oj} = H_{oj}(\bar{Y}_j,X) = H_j(\bar{Y}_j,X;\beta,\gamma_o)$.

To show double robustness, we show that when $\beta=\beta_o$, $E[\Psi(\beta,\alpha_o,\gamma_o)] = 0$. First we assume that the imputation model is correctly specified such that $H_{oj} = E_{Y_{j+1},\ldots,Y_D}(U(\beta)|\bar{Y}_j,R_j=1,X)$. First take expectation with regards to the first term of equation (I.1).

$$\begin{aligned}E_{R_1,\bar{Y}_D|X}\left(\frac{R_1}{\tilde{\pi}_{o1}}H_{o1}\right) &= E_{R_1|X}E_{Y_1|R_1,X}\left(\frac{R_1}{\tilde{\pi}_{o1}}E_{Y_2,\ldots,Y_D}(U(\beta)|Y_1,R_1=1,X)\right)\\
&= E_{R_1|X}\left(\frac{R_1}{\tilde{\pi}_{o1}}E_{Y_1}[E_{Y_2,\ldots,Y_D}(U(\beta)|Y_1,X)|X]\right) \quad \text{(I.2)}\\
&= E_{R_1|X}\left(\frac{R_1}{\tilde{\pi}_{o1}}E_{\bar{Y}_D}(U(\beta)|X)\right)\end{aligned}$$

Equation (I.2) hold because of f-MAR. It can be shown that $E_{\bar{Y}_D}(U(\beta)|X) = 0$ when $\beta=\beta_o$.

Next, for any $(j, \bar{Y}_{j-1}, \bar{R}_j, X)$, such that $2 \leqslant j \leqslant D$,

$$\begin{aligned}
&E_{Y_j|\bar{Y}_{j-1},\bar{R}_j,X}\left[\frac{R_j}{\tilde{\pi}_{oj}}(H_{oj} - H_{0,j-1})\right] \\
&= \frac{R_j}{\tilde{\pi}_{oj}}\left\{E_{Y_j}\left[E_{Y_{j+1},\ldots,Y_D}(U(\beta)|\bar{Y}_j, R_j = 1, X)\big|\bar{Y}_{j-1}, R_j = 1, X\right] - H_{0,j-1}\right\} \\
&= \frac{R_j}{\tilde{\pi}_{oj}}\left\{E_{Y_j,\ldots,Y_D}(U(\beta)|\bar{Y}_{j-1}, R_j = 1, X) - H_{0,j-1}\right\} && \text{(I.3)} \\
&= \frac{R_j}{\tilde{\pi}_{oj}}\left\{E_{Y_j,\ldots,Y_D}(U(\beta)|\bar{Y}_{j-1}, R_{j-1} = 1, X) - E_{Y_j,\ldots,Y_D}(U(\beta)|\bar{Y}_{j-1}, R_{j-1} = 1, X)\right\} && \text{(I.4)} \\
&= 0
\end{aligned}$$

where equation (I.3) and equation (I.4) hold because of f-MAR. Next, we assume the dropout model is correctly specified such that $\tilde{\pi}_{oj} = P(R_j = 1|\bar{Y}_{j-1}, X)$, $\forall j = \{1, \ldots, D\}$.

Using equation (6.14), taking expectation of the first component of the estimating equation:

$$\begin{aligned}
E_{\bar{R}_D,\bar{Y}_D|X}\left\{\frac{R_D}{\tilde{\pi}_{oD}}U(\beta)\right\} &= E_{\bar{Y}_D|X}E_{\bar{R}_D|\bar{Y}_D,X}\left\{\frac{R_D}{\tilde{\pi}_{oD}}U(\beta)\right\} \\
&= E_{\bar{Y}_D|X}\left\{\frac{1}{\tilde{\pi}_{oD}}U(\beta)P(R_D = 1|\bar{Y}_D, X)\right\} \\
&= E_{\bar{Y}_D|X}\left\{\frac{1}{\tilde{\pi}_{oD}}U(\beta)\tilde{\pi}_{oD}\right\} && \text{(I.5)} \\
&= E_{\bar{Y}_D}(U(\beta)|X) = 0 \quad (\text{when } \beta = \beta_o)
\end{aligned}$$

where equation (I.5) hold because of f-MAR. Finally in the second component of equation (6.14), for any $(j, \bar{Y}_D, X)$ such that $j \leqslant D - 1$,

$$\begin{aligned}
&E_{\bar{R}_D|\bar{Y}_D,X}\left\{\left(\frac{R_j}{\tilde{\pi}_{oj}} - \frac{R_{j+1}}{\tilde{\pi}_{o,j+1}}\right)H_{oj}\right\} \\
&= \left\{\frac{P(R_j = 1|\bar{Y}_D, X)}{\tilde{\pi}_{oj}} - \frac{P(R_{j+1} = 1|\bar{Y}_D, X)}{\tilde{\pi}_{o,j+1}}\right\}H_{oj} \\
&= \left\{\frac{\tilde{\pi}_{oj}}{\tilde{\pi}_{oj}} - \frac{\tilde{\pi}_{o,j+1}}{\tilde{\pi}_{o,j+1}}\right\}H_{oj} && \text{(I.6)} \\
&= 0
\end{aligned}$$

where equation (I.6) hold because of f-MAR.

Before we prove the rest of Theorem 2, we need the following proposition:

Proposition: Let X_{-D} be a vector of covariates which does not include D. Then, if independent death holds,

$$f(Y_s|R_j = 1, R_{j+1} = 0, \bar{Y}_j, D = l, D \geqslant s, X_{-D}) = f(Y_s|R_j = 1, R_{j+1} = 0, \bar{Y}_j, D \geqslant s, X_{-D}) \quad \text{(I.7)}$$

$\forall j < s < t$ and under (2), $\forall j < s < l$

Proof of Proposition:

First we note that independent death implies

$$P(D \geqslant t|D \geqslant s, Y_s, \bar{Y}_j, R_j = 1, R_{j+1} = 0, X_{-D}) = P(D \geqslant t|D \geqslant s, \bar{Y}_j, R_j = 1, R_{j+1} = 0, X_{-D}), \quad \forall j < s < t$$

The left-hand side of equation (I.7) can be written as

$$\begin{aligned}
&f(Y_s|R_j = 1, R_{j+1} = 0, \bar{Y}_j, D = l, D \geqslant s, X_{-D}) \\
&= f(Y_s|R_j = 1, R_{j+1} = 0, \bar{Y}_j, D \geqslant s, X_{-D}) \frac{P(D = l|D \geqslant s, Ys, \bar{Y}_j, R_j = 1, R_{j+1} = 0, X_{-D})}{P(D = l|D \geqslant s, \bar{Y}_j, R_j = 1, R_{j+1} = 0, X_{-D})} \\
&= f(Y_s|R_j = 1, R_{j+1} = 0, \bar{Y}_j, D \geqslant s, X_{-D}) \times \\
&\qquad \frac{P(D \geqslant l|D \geqslant s, Ys, \bar{Y}_j, R_j = 1, R_{j+1} = 0, X_{-D}) - P(D \geqslant l+1|D \geqslant s, Ys, \bar{Y}_j, R_j = 1, R_{j+1} = 0, X_{-D})}{P(D \geqslant l|D \geqslant s, \bar{Y}_j, R_j = 1, R_{j+1} = 0, X_{-D}) - P(D \geqslant l+1|D \geqslant s, \bar{Y}_j, R_j = 1, R_{j+1} = 0, X_{-D})} \\
&= f(Y_s|R_j = 1, R_{j+1} = 0, \bar{Y}_j, D \geqslant s, X_{-D}) \quad \text{(by independent death)}
\end{aligned}$$

Rest of Theorem 2: The dropout model and the imputation model need not condition on D if the following conditions are met: (1) u-MAR holds, and the dropout model is correctly specified, or if (2) f-MAR, p-MAR and independent death hold, and $H_j(\bar{Y}_j, X; \beta, \gamma)$ is correctly specified. Note that p-MAR implies mortal-cohort dDTIC.

Again, to show double robustness, we show that when $\beta = \beta_o$, $E[\Psi(\beta, \alpha_o, \gamma_o)] = 0$. First we assume that the imputation model is correctly specified such that $H_{oj} = E_{Y_{j+1},\ldots,Y_D}(U(\beta)|\bar{Y}_j, R_j = 1, X)$. Again, let $U_s(\beta)$ be the sth component of $U(\beta)$. First take expectation with regards to the first term of equation (I.1).

$$\begin{aligned}
E_{R_1,\bar{Y}_D|X}\left(\frac{R_1}{\tilde{\pi}_{o1}}H_{o1}\right) &= E_{R_1,Y_1|X}\left(\frac{R_1}{\tilde{\pi}_{o1}}E_{Y_2,\dots,Y_D}(U(\beta)|Y_1,R_1=1,X)\right) \\
&= E_{R_1|D\geqslant s}\left(\frac{R_1}{\tilde{\pi}_{o1}}E_{Y_1}\left[E_{Y_2,\dots,Y_D}\left(\sum_{s=1}^{D}U_s(\beta)|Y_1,D\geqslant s,X_{-D}\right)\Big|D\geqslant s,X_{-D}\right]\right) \qquad \text{(I.8)} \\
&= E_{R_1|D\geqslant s}\left(\frac{R_1}{\tilde{\pi}_{o1}}E_{\bar{Y}_D}\left[\sum_{s=1}^{D}U_s(\beta)|D\geqslant s,X_{-D}\right]\right) = 0 \quad (\text{when } \beta=\beta_o)
\end{aligned}$$

We now show equation (I.8) holds because of f-MAR, independent death, and p-MAR. For any sth component of $U(\beta)$,

$$\begin{aligned}
E_{Y_s}(U(\beta)|Y_1,R_1=1,X) &= E_{Y_s}(U(\beta)|Y_1,R_1=1,R_2=0,X) && \text{(I.9)} \\
&= E_{Y_s}(U(\beta)|Y_1,R_1=1,R_2=0,D\geqslant s,X_{-D}) && \text{(I.10)} \\
&= E_{Y_s}(U(\beta)|Y_1,D\geqslant s,X_{-D}) && \text{(I.11)}
\end{aligned}$$

Equation (I.9) holds because of f-MAR, equation (I.10) holds because of independent death, and equation (I.11) holds because of p-MAR.

Next, for any $(j,\bar{Y}_{j-1},\bar{R}_j,X)$, such that $2\leqslant j\leqslant D$,

$$\begin{aligned}
&E_{Y_j|\bar{Y}_{j-1},\bar{R}_j,X}\left[\frac{R_j}{\tilde{\pi}_{oj}}(H_{oj}-H_{o,j-1})\right] \\
&= \frac{R_j}{\tilde{\pi}_{oj}}\left\{E_{Y_j|\bar{Y}_{j-1},\bar{R}_j,X}E_{Y_{j+1},\dots,Y_D}\left(\sum_{s=1}^{D}U_s(\beta)|\bar{Y}_j,R_j=1,X\right)-H_{o,j-1}\right\} \\
&= \frac{R_j}{\tilde{\pi}_{oj}}\left\{E_{Y_j}\left[E_{Y_{j+1},\dots,Y_D}\left(\sum_{s=1}^{D}U_s(\beta)|\bar{Y}_j,R_j=1,R_{j+1}=0,X\right)\Big|\bar{Y}_{j-1},R_{j-1}=1,R_j=0,X\right]-H_{o,j-1}\right\} && \text{(I.12)} \\
&= \frac{R_j}{\tilde{\pi}_{oj}}\left\{E_{Y_j}\left[E_{Y_{j+1},\dots,Y_D}\left(\sum_{s=1}^{D}U_s(\beta)|\bar{Y}_j,R_j=1,R_{j+1}=0,X_{-D},D\geqslant s\right)\Big|\bar{Y}_{j-1},R_{j-1}=1,R_j=0,X_{-D},D\geqslant s\right]-H_{o,j-1}\right\} && \text{(I.13)} \\
&= \frac{R_j}{\tilde{\pi}_{oj}}\left\{E_{Y_j,\dots,Y_D}\left(\sum_{s=1}^{D}U_s(\beta)|\bar{Y}_{j-1},X_{-D},D\geqslant s)-H_{o,j-1}\right)\right\} && \text{(I.14)} \\
&= \frac{R_j}{\tilde{\pi}_{oj}}\left\{E_{Y_j,\dots,Y_D}\left(\sum_{s=1}^{D}U_s(\beta)|\bar{Y}_{j-1},X_{-D},D\geqslant s\right)-E_{Y_j,\dots,Y_D}\left(\sum_{s=1}^{D}U_s(\beta)|\bar{Y}_{j-1},X_{-D},D\geqslant s\right)\right\} && \text{(I.15)} \\
&= 0
\end{aligned}$$

Equation (I.12) holds because of f-MAR, equation (I.13) holds because of independent death, and equation (I.14) holds because of p-MAR. Similar arguments can be made for the second expectation in equation (I.15).

Next, we assume the dropout model is correctly specfied such that $\tilde{\pi}_{oj} = P(R_j = 1|\bar{Y}_j, X) = P(R_j = 1|\bar{Y}_{j-1}, D \geqslant j)$, $\forall j = \{1, \ldots, J\}$.

Using equation (6.14), taking expectation of the first component of the estimating equation:

$$\begin{aligned} E_{\bar{R}_D, \bar{Y}_D|X}\left\{\frac{R_D}{\tilde{\pi}_{oD}}U(\beta)\right\} &= E_{\bar{Y}_D|X}E_{\bar{R}_D|\bar{Y}_D,X}\left\{\frac{R_D}{\tilde{\pi}_{oD}}U(\beta)\right\} \\ &= E_{\bar{Y}_D|X}\left\{\frac{1}{\tilde{\pi}_{oD}}U(\beta)P(R_D = 1|\bar{Y}_D, X)\right\} \\ &= E_{\bar{Y}_D|X}\left\{\frac{1}{\tilde{\pi}_{oD}}U(\beta)\tilde{\pi}_{oD}\right\} \\ &= 0 \quad (\text{when } \beta = \beta_o) \end{aligned} \tag{I.16}$$

where equation (I.16) hold because of u-MAR.

Finally in the second component of equation (6.14), for any $(j, \bar{Y}_D, X)$ such that $j \leqslant D - 1$,

$$\begin{aligned} &E_{\bar{R}_D|\bar{Y}_D,X}\left\{\left(\frac{R_j}{\tilde{\pi}_{oj}} - \frac{R_{j+1}}{\tilde{\pi}_{o,j+1}}\right)H_{oj}\right\} \\ &= \left\{\frac{P(R_j = 1|\bar{Y}_{j-1}, D \geqslant j, X_{-D})}{\tilde{\pi}_{oj}} - \frac{P(R_{j+1} = 1|\bar{Y}_D, D \geqslant j+1, , X_{-D})}{\tilde{\pi}_{o,j+1}}\right\}H_{oj} \\ &= \left\{\frac{\tilde{\pi}_{oj}}{\tilde{\pi}_{oj}} - \frac{\tilde{\pi}_{o,j+1}}{\tilde{\pi}_{o,j+1}}\right\}H_{oj} \\ &= 0 \end{aligned} \tag{I.17}$$

where equation (I.17) hold because of u-MAR.

J. Supplementary simulation results

Table J.1: Misspecified dropout or imputation models in AIPW for simulation 1. AIPWd refers to AIPW with incorrect dropout models (sex is omitted), AIPWi refers to AIPW with incorrect imputation models (sex is omitted), and AIPWdi refers to AIPW with incorrect dropout and imputation models (sex is omitted in both models).

	AIPWd$_f$ (strat on D)			AIPWi$_f$ (strat on D)			AIPWdi$_f$ (strat on D)		
	bias	s.bias	SE	bias	s.bias	SE	bias	s.bias	SE
β_0	-0.003	-1.695	0.151	-0.003	-1.695	0.151	-0.003	-1.695	0.151
β_1	-0.003	-2.199	0.149	-0.004	-3.005	0.149	-0.002	-1.161	0.149
β_2	-0.008	-3.833	0.221	-0.010	-4.429	0.223	-0.044	-20.155	0.217
β_3	-0.009	-2.883	0.309	-0.012	-3.680	0.315	-0.109	-36.959	0.296
β_4	-0.011	-2.652	0.412	-0.010	-2.280	0.428	-0.084	-22.717	0.372
β_{sex}	0.008	3.500	0.217	0.008	3.500	0.217	0.008	3.500	0.217
β_{sex1}	-0.006	-2.897	0.207	-0.004	-2.027	0.207	-0.002	-1.232	0.206
β_{sex2}	-0.005	-1.722	0.315	-0.002	-0.588	0.317	0.016	4.958	0.312
β_{sex3}	0.006	1.337	0.411	0.010	2.446	0.417	0.068	17.135	0.400
β_{sex4}	0.005	0.947	0.562	0.005	0.936	0.575	0.050	9.533	0.528

Table J.2: Results under simulation 3 with true parameter values: $\beta_0 = 17.630, \beta_1 = 0.358, \beta_2 = 0.064, \beta_3 = -0.596, \beta_4 = -1.140$

	IEE			IPW$_u$			IPW$_p$			IPW$_f(D \in X)$		
	bias	s.bias	SE	bias	s.bias	SE	bias	s.bias	SE	bias	s.bias	SE
β_0	-0.0013	-1.2940	0.1039	-0.0013	-1.2940	0.1039	-0.0013	-1.2940	0.1039	-0.0013	-1.2940	0.1039
β_1	0.1717	151.0461	0.1137	0.0096	8.9202	0.1079	0.0096	8.9202	0.1079	-0.0036	-3.3368	0.1093
β_2	0.4285	266.4189	0.1608	0.0824	52.3295	0.1575	0.0918	58.1518	0.1579	0.0009	0.4966	0.1809
β_3	0.3442	165.6292	0.2078	-0.0289	-14.0999	0.2049	0.0221	10.8535	0.2036	0.0015	0.6655	0.2204
β_4	0.4229	157.7189	0.2682	-0.1025	-35.8201	0.2861	-0.0067	-2.4082	0.2785	-0.0079	-2.8390	0.2774

	AIPW$_u$			AIPW$_f(D \in X)$			MI$_u$			MI$_f(D \in X)$		
	bias	s.bias	SE	bias	s.bias	SE	bias	s.bias	SE	bias	s.bias	SE
β_0	-0.001	-1.294	0.104	-0.001	-1.294	0.104	-0.001	-1.294	0.104	-0.001	-1.294	0.104
β_1	0.010	9.115	0.108	-0.003	-2.864	0.108	0.008	7.305	0.108	-0.005	-4.762	0.109
β_2	0.095	61.808	0.154	0.001	0.476	0.162	0.096	62.352	0.154	0.005	3.116	0.157
β_3	0.038	19.243	0.199	0.000	-0.168	0.205	0.023	11.953	0.195	0.017	8.381	0.200
β_4	0.023	8.158	0.286	-0.006	-2.328	0.271	-0.012	-4.689	0.264	0.018	6.675	0.267

Table J.3: Simulation 3: methods stratifying on D

	IPW$_f$			AIPW$_f$			MI$_f$		
	bias	s-bias	SE	bias	s-bias	SE	bias	s-bias	SE
β_0	-0.001	-1.2940	0.104	-0.001	-1.294	0.104	-0.001	-1.294	0.104
β_1	-0.003	-2.743	0.110	-0.003	-2.765	0.109	-0.001	-1.037	0.108
β_2	0.003	1.724	0.181	0.000	-0.034	0.165	0.002	0.995	0.163
β_3	-0.004	-1.755	0.234	-0.001	-0.334	0.207	-0.008	-4.006	0.206
β_4	-0.007	-2.408	0.278	-0.006	-2.195	0.271	-0.024	-9.138	0.268

K. Analysis from a simulated data set

To reproduce the results found from the data analysis, we simulated a data set with similar design as the real data used in the paper (code on `https://github.com/lw499/mortalcohort_github`). The results from the analysis can be found on Table K.1.

Table J.4: Bias (×100), standardised bias (s-bias) and standard error (×100) from simulation 1 (N=1000). Note that method (1), D in modelled as a covariate, in method (2), D is stratified on

Parameter†	$\beta_1 = 0.698$			$\beta_2 = 0.962$			$\beta_3 = 0.567$			$\beta_4 = -0.162$			$\beta_{sex1} = -0.535$			$\beta_{sex2} = -1.087$			$\beta_{sex3} = -1.673$			$\beta_{sex4} = -1.248$		
	bias	s-bias	SE	bias	s-bias	SE	bias	s-bias	SE	bias	s-bias	SE	bias	s-bias	SE	bias	s-bias	SE	bias	s-bias	SE	bias	s-bias	SE
IEE	16.2	134.6	12.1	31.9	187.2	17.0	35.0	159.3	22.0	45.3	154.8	29.3	1.5	8.7	17.1	-4.6	-20.2	22.5	-6.2	-21.2	29.5	-3.9	-10.3	38.1
IPW$_u$	-0.1	-0.6	11.6	-0.2	-1.3	16.8	-0.6	-2.5	23.3	-0.6	-2.0	31.8	0.2	0.9	17.3	0.7	3.1	22.9	1.0	3.1	31.3	1.5	3.6	42.8
IPW$_p$	-0.1	-0.6	11.6	-0.2	-1.1	16.8	-0.6	-2.5	23.3	-0.9	-2.8	31.7	0.2	0.9	17.3	0.7	3.2	22.9	1.0	3.2	31.4	1.7	4.0	42.8
IPW$_f$ (1)	-0.1	-0.7	11.6	-0.2	-1.0	16.8	-0.5	-2.2	23.4	-0.8	-2.6	31.7	0.1	0.8	17.3	0.7	2.9	22.9	0.9	2.9	31.3	1.6	3.8	42.9
IPW$_f$ (2)	-0.1	-0.6	11.5	-0.1	-0.4	16.6	-0.4	-1.8	23.2	-0.9	-2.8	31.7	0.0	0.2	16.9	0.6	2.8	22.8	0.8	2.6	31.5	1.7	4.0	42.8
AIPW$_u$	-0.3	-2.4	11.1	-0.4	-2.6	16.3	-0.6	-2.7	22.2	-0.9	-3.1	29.2	0.6	3.8	15.7	1.2	5.8	21.6	1.3	4.3	29.6	1.9	5.0	38.0
AIPW$_f$(1)	-0.2	-2.1	11.0	-0.3	-2.0	16.1	-0.5	-2.4	22.0	-0.7	-2.5	29.3	0.5	3.0	15.6	1.1	5.2	21.5	1.2	4.1	29.5	1.7	4.6	38.0
AIPW$_f$(2)	-0.2	-2.0	11.0	-0.3	-1.8	16.1	-0.5	-2.0	22.1	-0.8	-2.6	29.4	0.4	2.7	15.5	1.0	4.7	21.4	1.1	3.9	29.5	1.8	4.6	38.1
L$_u$	-0.3	-2.4	11.1	-3.8	-23.6	16.0	-10.2	-48.0	21.3	-17.3	-61.9	27.9	0.6	4.0	15.6	1.5	7.1	21.2	5.0	17.7	28.4	6.7	19.0	35.5
LI$_f$(1)	-0.3	-3.1	11.0	-1.5	-9.2	15.9	-0.5	-2.4	21.2	-1.0	-3.4	28.1	0.6	3.8	15.5	1.8	8.7	21.1	1.7	6.1	28.4	2.1	6.0	35.6
LI$_f$(2)	-0.4	-3.5	11.0	-0.7	-4.5	16.0	-1.2	-5.4	21.5	-1.0	-3.5	28.5	0.6	3.9	15.5	1.6	7.5	21.3	2.0	7.0	28.9	2.4	6.5	36.4
MI$_u$	0.3	2.8	11.1	-3.6	-22.4	16.0	-9.8	-45.9	21.3	-16.9	-60.6	27.9	0.7	4.6	15.6	1.8	8.4	21.3	5.3	18.6	28.5	7.0	19.5	35.7
MI$_f$(1)	0.2	2.0	11.0	-1.3	-8.1	16.0	-0.1	-0.6	21.3	-0.6	-2.3	28.2	0.7	4.4	15.5	2.1	9.9	21.3	2.0	6.9	28.5	2.4	6.6	35.8
MI$_f$(2)	-0.3	-2.9	11.0	-0.5	-3.1	16.0	-0.7	-3.3	21.6	0.0	-0.2	28.7	0.6	3.9	15.5	1.6	7.7	21.3	2.1	7.3	29.0	2.5	6.7	36.7

†bias, standardised bias, and standard error for $\beta_0 = (0.1, 0.7, 10.2)$ and for $\beta_{sex} = (-0.2, -1.6, 14.6)$ in all methods

Table J.5: Bias (×100), standardised bias (s-bias) and standard error (×100) from simulation 2 (N=1000). Note that method (1), D in modelled as a covariate, in method (2), D is stratified on

Parameter‡	$\beta_1 = 0.698$			$\beta_2 = 0.872$			$\beta_3 = 0.354$			$\beta_4 = -0.414$			$\beta_{sex1} = -0.535$			$\beta_{sex2} = -1.095$			$\beta_{sex3} = -1.616$			$\beta_{sex4} = -1.232$		
	bias	s-bias	SE	bias	s-bias	SE	bias	s-bias	SE	bias	s-bias	SE	bias	s-bias	SE	bias	s-bias	SE	bias	s-bias	SE	bias	s-bias	SE
IEE	17.2	149.0	11.6	37.3	223.0	16.7	49.1	232.1	21.2	58.9	216.6	27.2	-0.4	-2.6	16.4	-5.6	-24.4	22.9	-13.3	-47.6	28.0	-8.9	-25.2	35.1
IPW$_u$	1.0	9.1	11.3	4.0	23.8	16.8	12.0	54.9	21.9	10.7	35.6	29.9	-1.7	-10.0	16.7	0.5	2.2	24.1	-5.1	-16.8	30.0	-2.9	-7.5	39.5
IPW$_p$	1.0	9.1	11.3	3.2	19.0	16.9	10.2	45.3	22.5	16.9	54.4	31.1	-1.7	-10.0	16.7	-1.6	-6.7	24.3	-9.9	-31.9	30.9	-12.8	-31.4	40.7
IPW$_f$ (1)	0.6	5.3	11.3	-15.5	-81.6	19.0	0.5	2.0	25.4	24.2	85.8	28.2	-1.8	-10.7	16.8	6.9	25.0	27.7	-11.9	-33.9	35.1	-9.0	-23.9	37.5
IPW$_f$ (2)	-1.6	-13.9	11.6	3.9	22.7	17.1	-0.9	-3.7	24.0	16.9	54.4	31.1	0.6	3.5	16.8	-8.7	-35.2	24.7	-6.9	-21.2	32.7	-12.8	-31.4	40.7
AIPW$_u$	0.8	7.1	10.8	2.8	18.3	15.5	7.7	37.3	20.7	17.6	59.9	29.4	-1.2	-7.9	15.3	-1.0	-4.8	21.7	-4.3	-15.5	27.9	-11.3	-29.6	38.1
AIPW$_f$(1)	-0.3	-3.0	10.8	-1.6	-9.6	16.1	-5.9	-27.3	21.5	13.7	50.9	26.9	0.8	5.0	15.3	2.1	9.2	22.6	2.7	9.1	29.7	-7.6	-21.6	35.3
AIPW$_f$(2)	-3.1	-27.9	11.1	-0.5	-3.3	15.8	-5.6	-25.4	22.1	13.9	49.0	28.4	3.3	21.0	15.6	0.8	3.7	22.2	2.3	7.6	29.8	-7.9	-21.0	37.5
MI$_u$	0.7	6.8	10.8	0.1	0.5	15.3	0.6	2.9	19.8	1.7	6.6	25.9	-1.1	-7.5	15.2	-0.8	-3.6	21.2	-1.2	-4.6	26.4	-2.8	-8.4	33.8
MI$_f$(1)	-0.4	-3.7	10.8	-1.5	-10.0	15.4	-5.6	-27.1	20.6	13.4	51.4	26.0	0.9	5.7	15.2	1.3	6.3	21.2	3.2	11.9	26.9	-6.7	-19.9	33.8
MI$_f$(2)	-3.0	-27.4	11.0	-1.0	-6.5	15.6	-6.3	-29.0	21.8	13.4	50.7	26.4	3.1	19.9	15.4	1.4	6.8	21.5	3.1	10.7	28.7	-7.0	-20.5	34.3
LI$_u$	0.8	7.6	10.8	0.3	2.1	15.4	0.5	2.2	19.9	1.3	5.0	25.9	-1.1	-7.3	15.2	-0.7	-3.4	21.2	-1.2	-4.3	26.5	-2.7	-7.9	33.7
LI$_f$(1)	0.2	2.2	10.8	-1.4	-9.0	15.4	-5.5	-26.6	20.7	14.3	54.8	26.1	0.8	5.2	15.2	1.6	7.3	21.2	3.5	12.9	27.0	-7.0	-20.6	34.0
LI$_f$(2)	-3.1	-28.2	11.1	-1.1	-6.8	15.6	-7.2	-32.9	21.8	13.2	49.9	26.4	3.2	20.8	15.4	1.3	6.2	21.5	3.4	11.7	28.7	-7.1	-20.6	34.3

‡bias, standardised bias, and standard error for $\beta_0 = (-0.1, -1.1, 10.3)$ and for $\beta_{sex} = (0.2, 1.1, 14.8)$ in all methods

Table K.1: Parameter estimate (standard error) of a simulated data set similar to OCTO.

	$\hat{\beta}_0$	$\hat{\beta}_1$	$\hat{\beta}_2$	$\hat{\beta}_{sex}$	$\hat{\beta}_{age}$	$\hat{\beta}_{edu}$	$\hat{\beta}_{smo}$	$\hat{\beta}_{sex1}$	$\hat{\beta}_{age1}$	$\hat{\beta}_{edu1}$	$\hat{\beta}_{smo1}$
LMM	19.451 (0.350)	0.256 (0.080)	-0.063 (0.007)	-3.391 (0.233)	-0.159 (0.045)	0.161 (0.045)	-0.608 (0.231)	0.047 (0.043)	0.009 (0.008)	-0.006 (0.009)	-0.133 (0.043)
IEE	19.535 (0.392)	0.296 (0.107)	-0.067 (0.009)	-3.427 (0.240)	-0.157 (0.049)	0.144 (0.051)	-0.611 (0.239)	0.127 (0.060)	0.015 (0.012)	0.000 (0.013)	-0.170 (0.059)
IPW_u	19.600 (0.416)	0.132 (0.162)	-0.078 (0.018)	-3.421 (0.265)	-0.169 (0.058)	0.146 (0.053)	-0.651 (0.273)	0.161 (0.093)	0.035 (0.020)	0.002 (0.016)	-0.101 (0.090)
IPW_p	19.590 (0.410)	0.131 (0.154)	-0.076 (0.017)	-3.431 (0.261)	-0.169 (0.056)	0.144 (0.052)	-0.589 (0.269)	0.167 (0.090)	0.035 (0.018)	0.003 (0.015)	-0.135 (0.088)
IPW_f	19.610 (0.465)	0.228 (0.162)	-0.083 (0.019)	-3.418 (0.301)	-0.179 (0.056)	0.141 (0.057)	-0.583 (0.305)	0.139 (0.091)	0.029 (0.018)	0.003 (0.015)	-0.156 (0.090)
$AIPW_u$	19.573 (0.389)	0.299 (0.145)	-0.072 (0.017)	-3.475 (0.256)	-0.162 (0.054)	0.142 (0.052)	-0.547 (0.260)	0.082 (0.092)	0.013 (0.021)	0.003 (0.014)	-0.241 (0.089)
$AIPW_f$	19.560 (0.435)	0.264 (0.141)	-0.069 (0.016)	-3.462 (0.281)	-0.165 (0.052)	0.144 (0.056)	-0.506 (0.292)	0.063 (0.072)	0.014 (0.017)	0.003 (0.013)	-0.226 (0.073)
MI_u	19.511 (0.369)	0.277 (0.101)	-0.066 (0.010)	-3.478 (0.228)	-0.150 (0.047)	0.154 (0.049)	-0.575 (0.235)	0.087 (0.050)	0.007 (0.010)	-0.005 (0.011)	-0.203 (0.051)
MI_f	19.540 (0.370)	0.249 (0.105)	-0.065 (0.010)	-3.470 (0.230)	-0.151 (0.047)	0.149 (0.049)	-0.563 (0.237)	0.076 (0.050)	0.009 (0.011)	-0.003 (0.012)	-0.196 (0.052)

References

Aalen, O.O. and Gunnes, N. (2010). A dynamic approach for reconstructing missing longitudinal data using the linear increments model. *Biostatistics* **11**, 453–472.

Diggle, P., Farewell, D. and Henderson, R. (2007). Analysis of longitudinal data with drop-out: objectives, assumptions and a proposal. *Applied Statistics* **56**, 499–550.

Seaman, S.R. and Copas, A. (2009). Doubly robust generalized estimating equations for longitudinal data. *Statistics in Medicine* **28**, 937–955.

Seaman, S.R., White, I.R. and Farewell, D. (2016). Linear increments with non-monotone missing data and measurement error. *Scandinavian Journal of Statistics*.
